# Chitosan Hydrogel as Tissue Engineering Scaffolds for Vascular Regeneration Applications

**DOI:** 10.3390/gels9050373

**Published:** 2023-05-01

**Authors:** Qiulin Wang, Xiaoyu Wang, Yakai Feng

**Affiliations:** 1 School of Chemical Engineering and Technology, Tianjin University, Yaguan Road 135, Tianjin 300350, China; jiumo0925@163.com (Q.W.); wangxiaoyuly@163.com (X.W.); 2 Collaborative Innovation Center of Chemical Science and Chemical Engineering (Tianjin), Weijin Road 92, Tianjin 300072, China; 3 Key Laboratory of Systems Bioengineering, Ministry of Education, Tianjin University, Weijin Road 92, Tianjin 300072, China; 4 Frontiers Science Center for Synthetic Biology, Tianjin University, Weijin Road 92, Tianjin 300072, China

**Keywords:** chitosan, hydrogel, tissue engineering, vascular regeneration, angiogenesis

## Abstract

Chitosan hydrogels have a wide range of applications in tissue engineering scaffolds, mainly due to the advantages of their chemical and physical properties. This review focuses on the application of chitosan hydrogels in tissue engineering scaffolds for vascular regeneration. We have mainly introduced these following aspects: advantages and progress of chitosan hydrogels in vascular regeneration hydrogels and the modification of chitosan hydrogels to improve the application in vascular regeneration. Finally, this paper discusses the prospects of chitosan hydrogels for vascular regeneration.

## 1. Introduction

Over the last century, tissue engineering has emerged as a crucial research field and a hotspot in regenerative medicine, interweaving multiple disciplines such as life science, material science, and engineering. The field has yielded a plethora of tissue engineering products that are being employed in clinical applications to repair damaged tissues and organs [1]. With the increasing prevalence of vascular-related diseases, the development of tissue-engineered scaffold tailored for vascular reconstruction and regeneration is a pressing concern. The ideal scaffolds should simulate the extracellular matrix (ECM), provide support for cells, and induce tissue regeneration. Furthermore, these scaffolds must possess excellent properties in biocompatibility, biodegradability, mechanical performances, and bioactivities. For instance, tissue-engineered blood vessels and vascular scaffolds should mitigate the risk of thrombosis and immune rejection, while simultaneously exhibiting good biomechanical properties that can withstand blood pressure [2]. For microvascular regeneration, the micro-/macro-structure, porosity, and pro-angiogenic bioactivity of the scaffolds are particularly important.

Hydrogels serve as one of the most popular tissue engineering scaffold materials owing to their unique structure, excellent hydrophilicity, and biocompatibility. The three-dimensional network architecture of hydrogels resembles that of natural extracellular matrix, ensuring high water content and retention within the scaffold [3]. Hydrogels can especially also sustain release and deliver bioactive molecules, drugs, genes, and siRNAs, making them ideal candidates for tissue engineering strategies that require controlled and targeted delivery of therapeutic agents [4,5]. The synergistic combination of hydrogels and bioactive components has shown more conducive to the reconstruction of damaged tissues and organs, which makes it a highly attractive approach for the development of advanced tissue engineering therapies.

Chitosan has garnered increasing interest in the fields of biomedicine and tissue engineering due to its excellent biocompatibility and biological activities [6]. Meanwhile, chitosan boasts a range of advantages, including but not limited to adsorption, coagulation induction, anti-inflammatory properties, drug loading, and sustained release [7].

More interestingly, chitosan hydrogels fully combine the advantages of chitosan and hydrogel, allowing for the adaption to repair the damaged tissue microenvironment as well as to combine bioactive components [8,9]. With their high porosity, bioadhesive properties to cells, and other functionalities, chitosan hydrogels have become an ideal biomaterial that facilitates a wide range of applications in tissue engineering. Furthermore, they exhibit advantages in nanomedicines in drug delivery. They can achieve a controlled release of the drug and continuously promote angiogenesis at the damaged tissue [10]. The injectable chitosan hydrogels offer excellent shape adaptation while enabling in situ drug release and treatment. Their own affinity for cells combining with drug effect can promote the repair effect of tissue engineered scaffolds.

**Figure 1:**
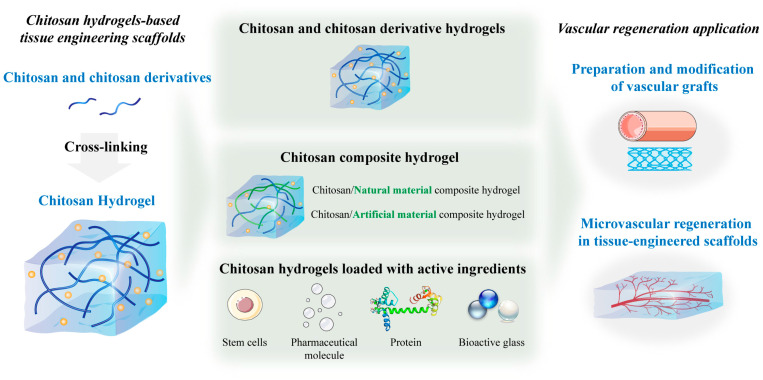
Chitosan hydrogels and their applications in vascular regeneration.

Focusing on chitosan hydrogels-based tissue-engineered scaffolds for vascular regeneration applications, this paper mainly introduces following aspects: (1) tissue engineering scaffolds for vascular regeneration, (2) technologies for the preparation of chitosan hydrogel scaffolds in vascular remodeling, (3) chitosan and its derivatives for vascular regeneration, (4) chitosan hydrogels and their applications in vascular remodeling. We also have introduced the modification of chitosan hydrogels by structural design and introducing bioactive substances for better vascular regeneration applications (Figure 1). Finally, the progress and prospects of chitosan hydrogels for vascular regeneration will be discussed to provide new ideas for future research in the field of tissue engineering scaffolds.

## 2. Tissue Engineering Scaffolds for Vascular Regeneration

Tissue engineering is dedicated to resolving the issues of immune rejection associated with allogeneic/xenograft and artificial organs, simultaneously avoiding the additional trauma incurred by autologous transplantation [11]. As one of the three primary elements of tissue engineering, scaffolds serve as pivotal frame materials to support tissue formation. In recent years, scaffolds have been widely used in bone [11,12,13], vessels [14,15,16,17], skin [18,19], nerve [20,21,22], cornea [23,24], tendon [25], and periodontal tissue engineering [26,27,28].

The repair, maintenance, and improvement of damaged tissues and organs by tissue engineering frequently necessitates the regeneration or remodeling of blood vessels in these tissues [29,30]. The vascular regeneration and remodeling benefit the repair of the structure and function of damaged tissues and organs. Vascular remodeling involves macrovascular and microvascular remodeling. For the remodeling of macrovascular vessels following the implantation of artificial vessel and vascular stents, extensive studies primarily focus on the performance of vascular grafts, such as the mechanical properties, anti-coagulation, anti-proliferation, and pro-endothelialization [29,30,31]. The interactions among implant materials, blood, endothelial cells (ECs), and smooth muscle cells (SMCs) play vital roles in macrovascular remodeling [29,32]. In particular, the design and fabrication of vascular grafts should aim to optimize the interactions between the implant materials and the host’s intra-biological environment to ensure the long-term success of these devices. In the case of bone tissue regeneration, wound healing, soft gingival tissue regeneration, and hindlimb ischemic repair, the microvascular remodeling within the scaffold plays a key role in the restoration of tissue function [33]. Consequently, the functional requirements for these scaffolds mainly highlight their pro-angiogenic activity, including angiogenesis, arteriogenesis, and vasculogenesis [34,35,36,37]. In order to improve the scaffold’s ability to support cells, a common research idea is to modify the scaffold surface to create an interface with specific biological functions [38,39,40]. Examples include drug-eluting scaffolds, surface modification of 3D fabricated scaffolds, hydrogel coating, and alteration of scaffold surface morphology.

Drug delivery systems are often utilized to mobilize cells or surrounding internal environments during microvascular remodeling [41,42]. Hydrogels offer an inherent advantage in this regard due to their water-insoluble 3D structure, which facilitates both cell encapsulation and drug loading delivery. The design and manufacture of tissue engineering scaffolds for vascular regeneration must take into account numerous performance requirements, such as good biocompatibility, suitable mechanical properties, biodegradability, plasticity, and sterilizability. This represents a common research direction aimed at improving the scaffold to support and regulate cells in vascular regeneration. In a word, tissue engineering scaffolds hold immense potential for vascular regeneration and remodeling, paving the way for the development of novel therapies.

## 3. Technologies for the Preparation of Chitosan Hydrogel Scaffolds in Vascular Remodeling

### 3.1. Macrovascular Remodeling Scaffolds

The characteristics of biocompatibility, mechanical properties, and surface properties are important requirements for tissue-engineered scaffolds applied to the reconstruction of blood vessels [43,44]. The interaction of the scaffold with the cells is well worth including in consideration to enhance the proliferation, migration, and differentiation of target cells [43,45,46]. To promote the mechanical and biological properties of chitosan hydrogel scaffolds for macrovascular remodeling, many strategies have emerged to optimize them subject to vascular regeneration, such as biological coating technology [47,48,49,50], electrospinning technology, and 3D-bio-printing technology to prepare the scaffolds with precise microstructure [44,46,51,52]. The advantages of these technologies can be drawn upon to compensate for the lack of pure chitosan and to promote vascular regeneration.

#### 3.1.1. Biological Coating Technology

Biological coating is a surface modification method for scaffolds to adjust the interaction between material and cell/tissue by changing the surface properties, so as to improve the blood and cell compatibility of scaffolds [53,54,55,56]. The vascular biomaterials with anti-protein adhesion properties are conductive to modulate platelet responses and induce active endothelium [56].

In general, in the context of vascular stents, drug-eluting stents (DES) confer several clinical advantages over bare metal stents (BMS), mainly attributable to their surface coating and drug loading capabilities. The surface modified stents exhibit superior performance compared to the unmodified ones [57,58]. In order to enhance the endothelialization of medical devices in situ, our group have developed a matrix metalloproteinases (MMP) response surface, which can in situ smartly release gene and effectively enhance the proliferation and migration of ECs on the surface [57]. Moreover, the bioactive molecules such as therapeutic drugs and specific peptides can also promote endothelialization and blood compatibility [58].

In the realm of surface modification for DES, chitosan-based composite coatings have emerged as a promising approach to form the biointerface on metallic medical devices. Chitosan hydrogels can be easily fabricated into a film, and then the biological properties of the film can be further enhanced by coupling with other biomolecules or nano-components [59]. By studying the growth kinetics of structured hydrogels, researchers have successfully assembled multilayer hydrogels of chitosan with the help of electrodeposition. These hydrogels can function as a multifunctional platform for the co-deposition of biological components, enriching their structure and biological function and advancing the development of bionic tissue engineering [60].

Among the biological coating technologies, layer-by-layer self-assembly (LBL) has been demonstrated to be a simple and versatile method for surface modification. The polyelectrolytes are deposited alternately, creating a stable thin film on the surface of scaffolds with certain biological functions [61,62]. The mild LBL deposition conditions are conducive to maintaining the bioactivity of drugs. The biodegradability of chitosan allows for the slow and controlled release of the loaded drugs in vivo. Chitosan hydrogels can also enhance the biocompatibility of the interface while simultaneously serving as an excellent drug loading material. This bio-coating technology has been received considerable attention in recent years, particularly in the vascular scaffolds.

#### 3.1.2. Electrospinning Technology

Electrospinning is a vital technique for manufacturing the small diameter artificial blood vessels. In our previous work, gelatin-heparin layer and polyurethane layer were deposited continuously on a rotating mandrel-type traps by electrospinning technology to make the artificial blood vessels mimicking the morphological and mechanical properties of natural blood vessels [63,64]. In addition, we improved the hydrophilicity and blood compatibility of the scaffold by grafting hydrophilic polymer chains [65]. The electrospun scaffolds have a porous network structure mimicking ECM, have a large, specific surface area and stable mechanical properties, and are more suitable for cell growth and adhesion [66,67,68,69]. With the development of nanotechnology and gene delivery, electrospinning has become one of the best choices for the preparation of artificial blood vessels in recent years [70,71,72,73,74,75]. We used the electrospraying technology to load plasmid complexes on the electrospun scaffold, which can promote the growth of human umbilical vein endothelial cells and inhibit the proliferation of human umbilical artery smooth muscle cells [72,73]. Moreover, anticoagulant peptides and cell adhesive peptides have demonstrated advantages for the surface modification of the electrospun scaffolds [76,77,78,79]. These peptides enable the surface with low thrombosis and rapid endothelialization synergistically [77,78]. The low-fouling PEG and functional peptides could improve vascular endothelialization and long-term performance of vascular grafts [79,80].

The hydrogel-based nanofibrous membrane combines the advantageous properties of both fibrous membrane and hydrogels. The fibrous membranes significantly enhance the mechanical properties of chitosan hydrogels and elongate the drug release duration [81]. Additionally, chitosan hydrogels can encapsulate cells on the electrospun fibrous scaffolds, providing a conductive environment for cell survival and growth [82]. As a result, the hydrogel-based fibrous membranes exhibit excellent mechanical properties, sustained drug release, adhesion, antibacterial properties, and biocompatibility.

#### 3.1.3. 3D Printing Technology

Traditional technologies are insufficient for constructing the vascular scaffolds with precise shape and multifunctionality. The 3D printing technology offers the ability to customize the required biomechanical properties while effectively controlling the pore size and pore structure of vascular scaffolds. This technology is particularly well-suitable for making the scaffolds with complex structure and cells [83,84]. The bioprinted chitosan hydrogel offers a promising means of interfacial fixation for tissue engineering. By constructing a dual-scale porous network structure of hydrogels directly on the surface using 3D printing, the biologically fixed interface can support cell proliferation and adhesion [85].

### 3.2. Microvascular Remodeling Scaffolds

In the field of research on bone/chondral and skin tissue, several issues are often faced: fillability of irregular defects, minimally invasive treatment, microvascular remodeling of the surrounding environment, healing of wounds, etc. [86]. To address these issues, injectable scaffolds have been developed from the flowable sol-gels and have subsequently become a mainstream technology in the relevant fields. Chitosan hydrogel scaffolds have been investigated with a focus on pro-angiogenic activity for microvascular remodeling. Injectable scaffolds are accompanied by loading therapeutic agents, which can accelerate the microvascular regeneration process.

The injectable scaffold is a highly adaptable and shape-adjustable scaffold that shows promising applications in bone tissue and ischemic lower limbs, etc. [87,88]. Among them, hydrogels exhibit high water content and exceptional flexibility and are three-dimensional polymer networks with good biocompatibility, which are crucial for regulating cell function, maintaining tissue structure, and diffusing nutrients [89,90]. Hydrogels can be prepared by physical and chemical crosslinking and are commonly used as injectable materials. These hydrogel scaffolds find extensive applications in various fields such as optical and fluid actuators, tissue engineering scaffolds, cell culture substrates, and drug delivery carriers [91,92,93].

Hydrogels have been considered as a promising artificial matrix, and chitosan is among the most attractive components in the realm of injectable hydrogel research, owing to its vast array of applications in the biomedical field [94]. Injectable chitosan hydrogels incorporating nanoparticles can effectively improve the mechanical strength and biological properties, thereby conferring therapeutic effects in situ [95,96]. This also leads to the bright performance of chitosan hydrogels in the fields of cell encapsulation, drug delivery, and tissue repair.

### 3.3. Other Common Technologies in Vascular Remodeling

Chitosan hydrogels have many advantages, but the lack of mechanical strength remains a significant challenge. To overcome this limitation, researchers have explored several methods, including adjusting the composition and the preparation process, to enhance the scaffold strength [97,98,99]. The integrated preparation of tissue engineering scaffolds can be realized by computer simulation in advance [100,101]. The complexity can improve the scaffold stability [102,103]. Contemporary technology is often a variety of means to learn from each other, combining with the advantages of various processes to constantly optimize the preparation of tissue engineering scaffolds, in order to obtain scaffold materials with better performance. Table 1 provides an overview of the common techniques for tissue engineering scaffolds. We summarize these techniques here in order to well understand their features in vascular regeneration.

## 4. Chitosan and Its Derivatives for Vascular Regeneration

Chitosan is a biomaterial derived from chitin [114]. Chitosan derivatives with different properties are obtained through relevant modification, which helps to improve its solubility and broaden its biological applications [115,116]. The applications of chitosan and its derivatives in related fields are summarized in Table 2.

Chitosan possesses inherent antibacterial and coagulation-promoting abilities, making it a traditional biomaterial for wound dressing [117,118,119]. Chitosan hydrogel shows slow drug release profile, which attracts more attention in biomedicines [120,121,122]. However, its pro-coagulant biological function is disadvantageous in some situations for tissue engineering and vascular engineering application. Meanwhile, weak mechanical properties of chitosan hydrogels are also a major drawback for tissue engineering scaffold. In addition, they show a mismatching degradation rate, show low cell adhesion, and lack pro-therapeutic effects in vascular regeneration [123]. There are differences in the mechanical properties of chitosan hydrogels compared to natural organs [124]. The solubility of chitosan is also an issue in the application. Its insolubility in water and solubility in weak acid solutions largely limits its application. Fortunately, chitosan has many active amino and hydroxyl groups available for chemical modification to change its solubility, such as quaternization to enhance its antibacterial activity [125] and acidification to enhance its anticoagulation function [126], etc. In addition, adjusting the structure and composition of chitosan hydrogels also enables them with suitable performance and multifunction.

**Table 2:** Chitosan and its derivatives as well as their applications.

Modification Method	Material	Characteristic	Application	Producers’ Company	Specifications
Chitin deacetylated	Chitosan	Odorless and non-toxic solid, mostly white or off-white flake or powder, containing amino polysaccharide, soluble in aqueous acetic acid	Pharmaceutical active ingredient [127,128,129]; Drug dispersant [130,131]; Drug requests [132,133]	Shanghai Macklin’s Reagent Co. Shanghai Aladdin Biochemical Technology Co. etc.	Degree of deacetylation: 85%, 90%, 95%, 98% Viscosity (mPa·s): High (>400), Medium (200–400), Low (<200)
Chitosanase degradation	Chitosan oligosaccharide	Oligosaccharide polymerized from less than 10 glucosamine.	Enhanced cartilage [134,135,136]; Antioxidant [137,138]; Strengthens the immune system [139,140,141]	Adamas Reagents Ltd. Shanghai Macklin’s Reagent Co. etc.	Purity: 3000 Molecular weight Chitosan oligosaccharide lactate (<2000 Molecular weight)
Chemical modification of chitosan	Carboxymethyl chitosan	Introduction of carboxymethyl, amphoteric polyelectrolyte, according to the substitution of carboxymethyl, three products can be obtained.	Hemostatic materials [142,143,144]; Wound healing dressing [145,146,147]; Tissue repair gel [148,149,150,151]	Tianjin Heowns Biochem Technology Co. Leanlong Bohua (Tianjin) Pharmaceutical Chemicals Co. Dalian Meilun Biotechnology Co. Shanghai Yuanye Biotechnology Co. etc.	Degree of deacetylation: 85% Viscosity (mPa·s): 10–80 Carboxylation: ≥80%
	Hydroxypropyl chitosan	Hydroxypropyl group weakens the intermolecular and intramolecular hydrogen bonds of chitosan, destroys the spatial structure of chitosan, and enhances its water solubility and reactivity.	Increased healing tension [152] and moisturizing [153,154]	Shanghai Macklin’s Reagent Co. Shanghai Meryer Biochemical Technology Co. etc.	Substitution: ≥80%
	Quaternized chitosan	Introducing quaternary ammonium group or directly grafted small molecular quaternary ammonium salt to chitosan-NH_3_ active group.	Antiseptic bacteriostasis [155]; DNA carrier [156,157,158]; Composite film material [159,160,161]	Shanghai Macklin’s Reagent Co. Shanghai Yuanye Biotechnology Co. etc.	Substitution: 90–98%
	Chitosan sulfate	a modified product of SO4^2−^ group introduced into chitosan after vulcanization treatment. It is a polyamphoteric electrolyte, similar in structure to heparin.	Heparin substitutes [162,163,164,165]; Nonspecific immune enhancers [166,167,168]	-	-

### 4.1. Carboxymethyl Chitosan

**Figure 2:**
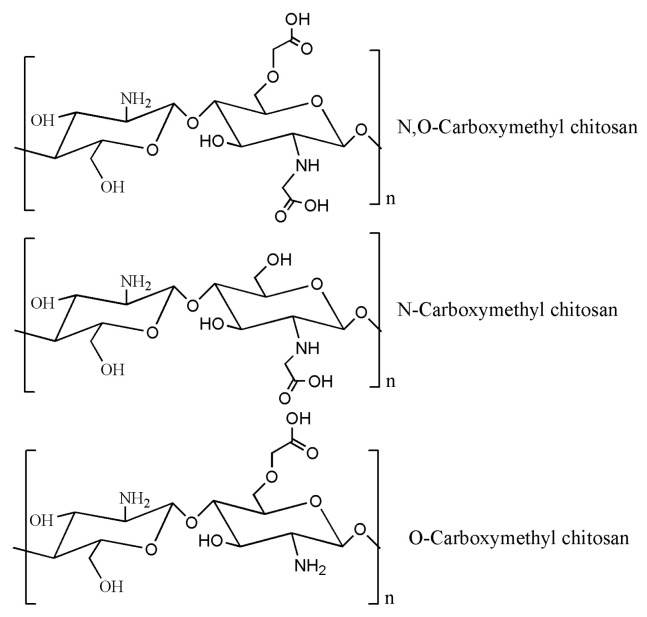
Chemical structures of N,O-carboxymethyl chitosan, N-carboxymethyl chitosan, and O-carboxymethyl chitosan. n represents the number of repeating units.

Carboxymethyl chitosan, as an early and widely researched modification of chitosan, has shown promising performance in promoting wound healing and tissue repair (Figure 2) [169]. Related studies have confirmed its ability to simulate the growth of fibroblasts, accelerate wound healing, and inhibit the proliferation of scar tissue [170,171]. Additionally, carboxymethyl chitosan also exhibits excellent film-forming property, as well as thickening of gel. It can prevent the adhesion of tissues and improve drug retention at the targeted site [172]. Its high viscosity and elasticity can prevent blood coagulation on tissue surface, thus promoting tissue repair [173]. Compared with chitosan, the carboxymethyl chitosan hydrogel can enhance the membrane penetration ability of biological macromolecule drugs, which contributes to good therapeutic effect [174].

### 4.2. Quaternary Ammonium Modified Chitosan

**Figure 3:**
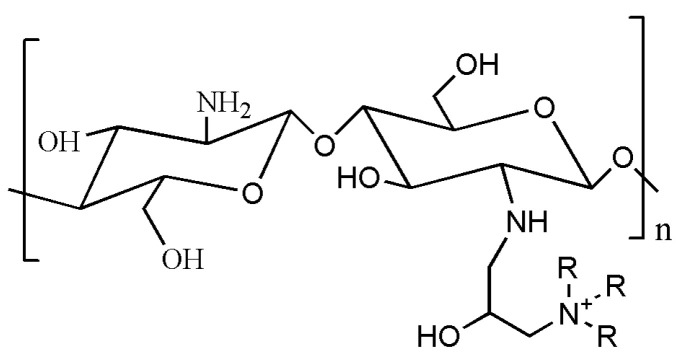
Chemical structure of quaternary ammonium chitosan. n represents the number of repeating units.

The quaternary-ammonium chitosan exhibits several advantages compared with chitosan such as better selective absorption of proteins and less dependence on solution pH (Figure 3) [125]. As a polycationic substance, it can combine with DNA to form the polymeric electrolyte complexes, making it suitable for gene targeting delivery [158]. Quaternary ammonium chitosan hydrogel is also effective in reducing sudden release, making it a promising protein drug carrier [160]. Moreover, it can also be used as a carrier for peptides and drugs with the long release period, thus enhancing the utilization rate of drugs.

### 4.3. Chitosan Sulfate

**Figure 4:**
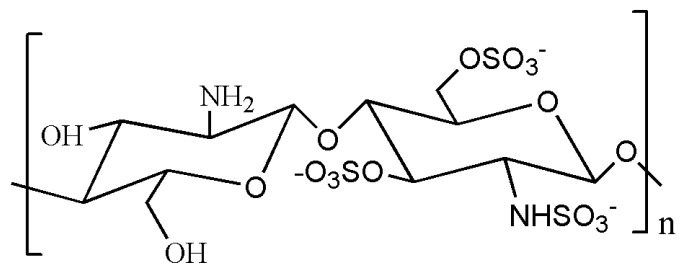
Chemical structure of chitosan sulfate. n represents the number of repeating units.

Chitosan sulfate is a modified form of chitosan prepared by introducing SO_4_^2−^ group through sulfuration and is endowed with a variety of physiological activities and functions (Figure 4) [163]. The chitosan sulfate has crucial biological properties like anticoagulation and antivirus, which is similar to heparin and chondroitin sulphate due to their shared sulphate structure [164]. In particular, chitosan sulfate boasts the strongest anticoagulant activity among chitosan derivatives. Therefore, the sulphating modification of chitosan has emerged as a pivotal focus in vascular tissue engineering research. The chitosan sulfate hydrogels can protect the negatively charged DNA from free radical attack and curb the generation of reactive oxygen species, thus minimizing damage to the body [175]. Notably, the substitution site and degree of deacetylation are important factors for its antioxidant performance [176,177]. The sulfated chitosan shows stronger antioxidant activity than other derivatives [178,179]. Chitosan sulphate hydrogels exhibit impressive swelling properties and also have the ability to bind to a variety of growth factors owing to their heparin-like structure [180,181].

## 5. Chitosan Hydrogels and Their Applications in Vascular Remodeling

Chitosan hydrogels are well known for their good biocompatibility owing to their composition and their network structure which closely resemble ECM [182,183,184]. Active chemical groups on chitosan may also enhance their biofunctions and performance, such as grafting functional groups with biological activities through chemical reactions which provides an exciting avenue for research in this field. The amino groups of chitosan hydrogels can form quaternary ammonium salt, which can enhance their antibacterial effect and strength property when combined with an anionic substance via electrostatic binding. In the above, we have briefly described chitosan derivatives as raw materials for the synthesis of chitosan hydrogels, from which some of the biological activity of chitosan hydrogels can be adjusted at source to meet the needs. Moreover, the structures of chitosan hydrogels can be regulated through various means to improve their plasticity, making them more conducive to the application of chitosan hydrogels in vascular repair and other aspects [183,185]. Vascularization is a crucial factor for tissue engineering and the long-term patency of vascular grafts. Vascularization also allows for a huge improvement in the internal microenvironment at the wound site, facilitating nutrient supply and transport and promoting wound healing [17]. In the case of angiogenic activity of vascular grafts and other implants requiring microvasculature, chitosan hydrogels often need to be augmented by drugs and bioactive substances. Enhancing vascularization is thus an important target for the functional improvement of chitosan hydrogels. In the following subsections, we will discuss chitosan and its derivative hydrogels, as well as composite hydrogels that are loaded with bioactive substances and cells, focusing on the methods and substances beneficial for rapid vascularization.

### 5.1. Chitosan and Its Derivative Hydrogels

As implants, chitosan hydrogels still face some fundamental problems, such as insufficient mechanical properties, low angiogenic activity, and low blood compatibility. To address these problems, we should develop the feasible approaches to improve and adjust the chemistry and structure of chitosan hydrogels via optimizing the crosslinkers, preparation methods, and domain modification [186]. Researchers have explored physical and chemical crosslinking methods to form a more stable three-dimensional network structure from chitosan. The physically crosslinked double network chitosan-polyvinyl alcohol (PVA) hydrogels were constructed through alternating freezing and heating treatments, which exhibited excellent compressibility (60%-230 KPa), stretchability (152 Kpa-360%), recoverability (77.5% after 90 cycles), and anti-swelling properties [187]. Given the abundance of hydroxyl and amino groups in chitosan’s macromolecular structure, it can be crosslinked with aldehydes and anhydrides. The most widely employed chemically crosslinking method with glutaraldehyde can create bridge structure that enable stable connections with the surrounding molecules [188]. Crosslinking agent plays an important role in the performance optimization of chitosan, as it can improve the material’s mechanical properties and surface properties. For instance, studies have shown that the surface modification of the crosslinked chitosan by phosphorylcholine significantly reduces the adsorption of proteins while maintaining the adsorption of bilirubin [189]. The efficient removal of the excess bilirubin in blood can facilitate the purification of blood through hemorfusion for hyperbilirubin treatment. In addition, the hydrogel has anticoagulant effect, which is of high value for the modification of chitosan hydrogels in medical fields that are in direct contact with blood, such as the cardiovascular field. There was a case study on the preparation of chitosan hydrogels using sodium tripolyphosphate or sodium hexametaphosphate as a crosslinking agent [190]. By adjusting the mass ratio of chitosan to crosslinking agent, the molecular structure of the crosslinked chitosan hydrogels can be controlled, and the dissolution/swelling behavior of the molecular chain segment can also be regulated, thus affecting the controlled release performance [191]. It is of great significance to design the structure of chitosan hydrogels or manipulate the strength of hydrogels through various techniques for tissue engineering angiogenesis. The above studies demonstrate that the choice of crosslinking agents or crosslinking methods can improve the properties of chitosan hydrogels.

Domain modification is another approach that can overcome the limitations of chitosan hydrogels in terms of bioactivity [192,193]. Zongjin Li and colleagues have demonstrated the efficacy of a thermo-responsive chitosan hydrogel incorporating the IGF-1C peptide for wound repair and angiogenesis [194]. IGF-1 is a potent mitogenic and pro-survival factor, and the use of its C structural domain facilitates cell migration and angiogenesis to modify the structure of chitosan hydrogels, avoiding the half-life problems associated with direct delivery of the relevant growth factors. This hydrogel has excellent tubular formation effect and was found to facilitate the regeneration of connective tissue, demonstrating its potential for therapeutic applications and making up for the lack of therapeutic effect.

**Figure 5:**
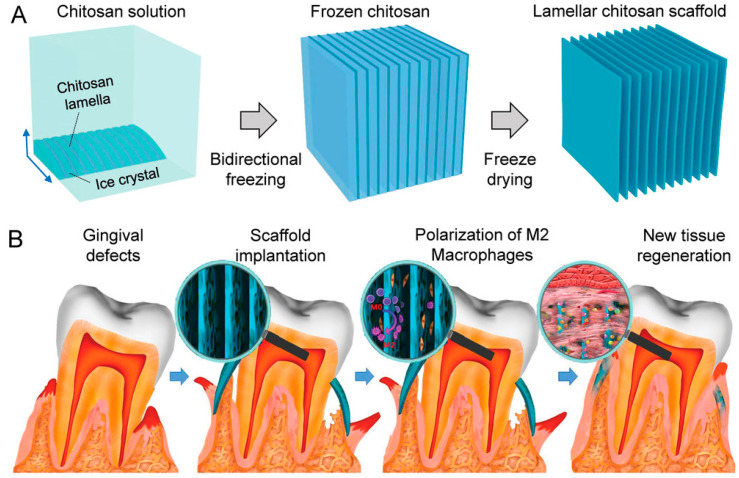
Schematic illustration of preparation and application of Lamellar Chitosan Scaffold (LCS). (**A**) Fabrication of LCS via a bidirectional freezing method. (**B**) An LCS was implanted as a gingival substitute for gingival regeneration through regulating macrophage polarization. Biomimetic lamellar chitosan scaffold for soft gingival tissue regeneration. Copyright 2021, Wiley [199].

The preparation process also provides the technical support for optimizing the macrostructure of chitosan hydrogel scaffolds and thus improving their performance. Three-dimensional printing is an emerging bio-manufacturing technology that can prepare highly complex specific scaffolds in the field of biomedicine [195]. Researchers have thereupon conducted extensive research in the use of 3D printing in the preparation of chitosan hydrogel scaffolds. It has been found that chitosan hydrogels have many limitations as inks for 3D printing. Therefore, we must modify chitosan to improve their 3D printing performance. Mina Rajabi et al. chemically modified the chitosan hydrogel through its large amount of amines and hydroxyl groups, facilitating formulation development [196]. Typically, organic solvents are used in the chitosan ink, which is contrary to the non-toxic and biocompatible characteristics of chitosan. To address this challenge, Luyu Zhou et al. directly printed high strength (compressive strength of 2.31 MPa) and complex chitosan hydrogels by dissolving chitosan in an alkaline solution [197]. The unique thermogenic gel properties of the ink make it possible to be printed by the embedded high-temperature solidification bath method. Inspired by natural organs and tissues, the hierarchical scaffolds with uniform interconnecting holes were manufactured by 3D printing technology, and they showed strong cell adhesion and infiltration [198]. Zuolin Wang and his team developed a bio-inspired layered chitosan scaffold (LCS) with a long-range ordered porous structure that mimics the gingival structure [199]. LCS can induce more blood vessel and fiber formation than conventional collagen membranes. The tensile strength of the hydrous LCS could reach up to approximately 15 MPa, and the toughness was 4 MJ/m^3^, which showed better mechanical properties than the commercial collagen membrane. Moreover, LCS was found to induce the macrophage differentiation into M2 macrophages, which can promote collagen synthesis (Figure 5). The 3D scaffold structure of LCS can effectively load different cells and drugs, making it a promising material for gingival tissue engineering, and it also creates a new idea for tissue replacement scaffolds in other fields. To achieve a biomimetic multilayer structure, Xiaowen Shi et al. fabricated layered hydrogels from stimulation-responsive amino chitosan by the electrical assembly process [200]. The equilibrium state between the electrophoretic speed of chitosan chain and the rate of diffused ion directly affects the structure of chitosan hydrogels. Among them, the anisotropic structure of chitosan hydrogels exhibited significantly increased mechanical strength: the modulus of elasticity increased from 0.22 MPa to 0.42 MPa, but the elongation at break decreased from 189.4% to 159.7%. Three-dimensional printing provides complex structures for chitosan hydrogels that can be closer to natural tissue structures, with more biologically robust and higher mechanical properties. This already shows that the ink modification and the structure construction of scaffolds with 3D printing technology are a trend for future related research.

Chitosan hydrogels also have an active presence in other technical areas. For instance, Amir Shamloo and colleagues prepared the double-layer vascular grafts with appropriate structure and biological properties using electrospinning and freeze-drying methods [201]. An anticoagulant inner layer (inner diameter 4 mm) composed of silk fibroin fiber and heparinized thermoplastic polyurethane co-electrospun fiber and an outer high-pore chitosan hydrogel were prepared by a freeze-drying method. This approach provided a non-thrombotic inner surface for endothelialization in the first few days and acted as a barrier to the migration of SMCs to the lumen [201]. Therefore, this graft offers potential applications as an artificial vascular scaffold for future in vivo transplantation.

In order to improve the properties of chitosan hydrogels, we can consider the microstructure and modification. In terms of microstructure, the crosslinking method and the crosslinking agents play the important roles. The advanced preparation technologies can also improve their properties and expand their applications. Additionally, the modification of chitosan hydrogels is also contributing to the development of tissue engineering owing to their multiple biofunctions.

### 5.2. Chitosan Composite Hydrogels

For chitosan hydrogels, researchers have improved their mechanical properties via structural optimization, changing the microscopic molecular structure and the macroscopic structure. Apart from these, their bioactivity and biocompatibility should also be improved by incorporating natural/artificial materials [202,203,204]. By combining other materials, the biocompatibility of chitosan hydrogels can be further increased, either by forming stable structure to improve their mechanical properties or by taking biological advantage of the combined materials to optimize the therapeutic effect in vascular regeneration. Composite hydrogels have the potential to overcome the limitations of each single component, and to amplify the biological effects.

#### 5.2.1. Chitosan/Natural Material Composite Hydrogels

Natural materials have their inherent biocompatibility and biofunctionality. Combined with them, the chitosan composite hydrogels are more biocompatible and facilitate the biological activity of natural materials. In the area of vascular regeneration, the hydrogel can be used to protect the natural active substances from degradation and destruction, thus enabling the biological advantages of the natural materials to be fully exploited and improving the therapeutic effect. Taking inspiration from the intricate layered structures and functions in natural biological systems, the chitosan composite hydrogels have been developed by integrating natural materials into the hydrogels to enhance their biological functions on the basis of bionic structure [205,206,207]. For instance, fibrin is known to promote the adhesion of arterial endothelial cells (ECs) and induce angiogenesis at the site of injury [207]. The interpenetrating polymer network of chitosan and fibrin contributes to the excellent self-healing and angiogenic capabilities, which compensates for the mechanical weaknesses and instability of fibrin gel. The composite hydrogel formed by hyaluronic acid and chitosan is injectable, biodegradable, and biocompatible, offering great advantages for the repair of full skin lesions. This highlights the advantages of chitosan composite hydrogels.

The loss of blood supply to any limb can result in ischemia, pain, and morbidity. To ensure proper blood perfusion and connection with surrounding tissues, the generation of functional microvasculature is an important topic in tissue engineering research. Utilizing an angiogenic agent to enhance blood flow to the limb can relieve pain and avoid amputation. Garry P. Duffy’s team investigated the combination of chitosan with β-glycerophosphates (β-GP) to form a heat-sensitive hydrogel (C/GP) [208]. The C/GP hydrogel increased the number of blood vessels in the thigh of the ischaemic limb about seven times. The hydrogel can be injected using standard needles and syringes, allowing for a more minimally invasive treatment for patients. The C/GP hydrogel is one of the most promising injectable scaffolds for carrying seed cells and promoting angiogenesis. The regeneration of blood vessels to ensure blood perfusion is a key of tissue engineering. Shan-hui Hsu’s team developed a chitosan fibrin-based self-healing hydrogel with a modulus of ~1.2 kPa, which enables vascular ECs to form capillary-like structure [209]. This hydrogel has shown promise in restoring the function of blood circulation system in patients with acute and chronic ischemic diseases, indicating its potential for future vascular repair application. Jingan Li et al. prepared the carboxymethyl chitosan/hyaluronate-dopamine (CMC/HA-DA) hydrogels via crosslinking by horseradish peroxidase and hydrogen peroxide with cell adhesion and antioxidant abilities [210]. CMC/HA-DA hydrogel can accelerate wound healing with high collagen deposition, granulation tissue, and skin appendage formation in vivo and can significantly promote angiogenesis and cell proliferation at the injury site.

Beyond combining other substances to form a more stable structure, chitosan hydrogels are also relevant as a kind of “bio-coating” due to their fluidity and adhesion properties. Chitosan hydrogels can modify the surface of cardiovascular stents, altering their surface properties and promoting their biocompatibility and blood compatibility. The surface modification can improve both anticoagulation and endothelialization of biomaterials. Hydrogel layer serves as an interface for biological functions in the field of blood contact materials, including blood compatibility and the growth of ECs while inhibiting the proliferation of SMCs. With the help of the chitosan hydrogels, the stable interface is ensured, and the biological action of the constituent components is also preserved. To this end, Changsheng Zhao and collaborators prepared a chitosan-κ-carrageenan composite hydrogel (C-K hydrogel) by an environmentally friendly method, and the introduction of carrageenan onto the surface of crosslinked chitosan hydrogels improved the biocompatibility, especially the anticoagulant properties [211]. The innovative hydrogel can be potentially used for stent modification and artificial blood vessel. In particular, the C-K hydrogel significantly inhibited the interactions of undesirable blood substances such as hemolysis, complement activation, platelet activation, and contact activation, thus demonstrating superior anticoagulant properties as compared to unmodified chitosan hydrogels. The optimized C-K hydrogels were even effective in reducing Escherichia coli by 46.0% and Staphylococcus aureus by 68.7%, respectively. Moreover, Lingren Wang et al. developed a multifunctional coated hydrogel thin films (HTFs) to modify vascular stents by LBL self-assembly and post-crosslinking of the sulfonated sodium alginate (SSA) and chitosan [212]. The HTF surface can inhibit the adsorption of blood protein, for example bovine serum fibrinogen (BFG) and also reduce platelet adhesion and activation. The HTFs improved the competitive growth of ECs while limiting the adhesion and proliferation of SMCs. The improved blood compatibility of the films is attributed to the introduction of various functional groups, while the strong selective effect on vascular cells is attributed to the synergistic effect of high sulphonation and the presence of the phenolic hydroxyl group. The unique HTFs exhibit excellent blood compatibility and high selectivity for vascular cells and may help guide the design of cardiovascular biomaterials for endothelialization.

#### 5.2.2. Chitosan/Artificial Material Composite Hydrogel

The biocompatibility and mechanical properties of hydrogels are the evaluation criteria for their application in tissue engineering. The development of chitosan-based hydrogels with desired mechanical strength is of great significance to the development of tissue engineering. The synthetic materials with desirable mechanical properties can be used to provide better mechanical properties for the composite hydrogel scaffolds. Composite hydrogels are perfectly suitable to a wide range of needs and can even be multifunctional at the same time.

The dual network (DN) structure is considered to be a promising strategy for increasing the mechanical properties of hydrogels [213]. This physical crosslinking allows both the effective absorption of mechanical energy without sacrificing the biocompatibility. It is also possible to simply adjust the mechanical strength of the DN hydrogels by adjusting the synthesis parameters. This hydrogel structure facilitates the effective functioning of the constituent components of the composite hydrogel. In a recent study, Xiguang Chen and collaborators prepared a high-strength ductile chitosan-PVA DN double-mesh hydrogel based on multiple hydrogen bond interactions. Subcutaneous implantation experiments in animals revealed the formation of a large number of new capillaries at the lesions, indicating the hydrogel’s potential to promote cell attachment [187]. In another study, Jinping Zhou et al. constructed a double crosslinked hydrogel of chitosan and polydopamine modified carbon nanotubes by the electrostatic interaction of phenylboric acid with the amino groups of chitosan, and the dynamic borate bond of phenylboric acid with the catecholic structure of the modified carbon nanotubes [214]. Carbon nanotubes (CNTs) complement chitosan hydrogel with excellent photothermal conversion properties, mechanical properties, and electrical conductivity. The presence of dopamine improved the dispersion and biocompatibility of carbon nanotubes, and chitosan promoted cell proliferation, angiogenesis, and granulation tissue formation. By combining multiple benefits, hydrogels exhibit biocompatibility, antioxidant properties, electrical conductivity, photothermal antibacterial activity, and in vivo hemostatic properties.

**Figure 6:**
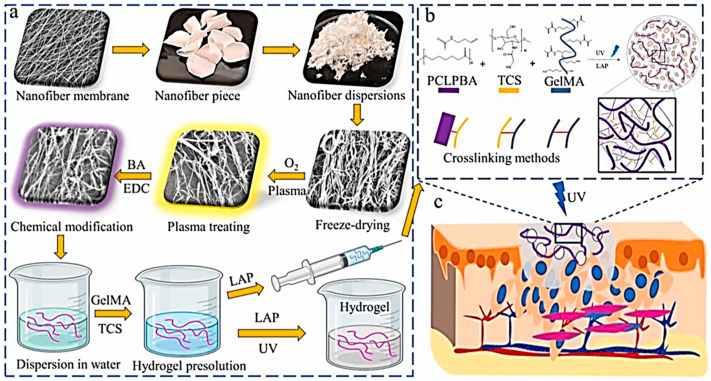
Schematic illustration of the hydrogel preparation. (**a**) Preparation of hydrogel and hydrogel precursor solution. (**b**) Chemical composition of the hydrogel precursor solution. The purple, yellow and blue lines indicate PCLPBA, TCS and GelMA, respectively. (**c**) Hydrogel formation in wound area. A multifunctional nanofiber reinforced photo-crosslinking hydrogel for skin wound healing. Copyright 2022, Elsevier [215].

The hydrogel designed to mimic the composition and structure of the natural ECM has been found to be more conducive to the adhesion and proliferation of functional cells. Xiumei Mo et al. prepared a photo-crosslinked hydrogel by simulating the structure and composition of natural fine ECM [215]. They designed nanofibrous hydrogels with the help of nanofibrous scaffolds, which have advantages in terms of structural similarity to natural ECM and high porosity, in combination with multifunctional photo-crosslinked chitosan hydrogels. It possessed rapid liquid-gel transition under ultraviolet (UV) exposure, excellent antibacterial effect, antioxidant property, and printability through 3D printing. The hydrogel can be compressed to 45% strain with a Young’s modulus of around 30 kPa and a medium network density to provide adequate mechanical strength. The hydrogel with nanofibers promoted cell immigration and facilitates angiogenesis by allowing cell penetration. When applied to full-thickness skin defects, the hydrogel can enhance blood vessel formation, epithelial regeneration, and wound healing (Figure 6). It is essential to promote epithelial formation for protecting the wound from serious infection during wound healing [216]. The hydrogel promotes endothelial growth and blood vessel formation, in the surrounding area, generating a functional microvasculature system that ensures appropriate blood perfusion and connects the wound to the surrounding tissues, thereby supplying nutrients needed for the wound to heal [209].

Furthermore, some artificial additives, such as nanoparticles and nanofibers, can also effectively enhance the mechanical properties of chitosan hydrogels [217]. These additives can act as delivery vehicles for drugs and bioagents, enabling targeted therapies and tissue engineering/repair [218]. Cheng Yang et al. designed a nanocomposite hydrogel with the ability to stimulate microangiogenesis due to polyhedral oligomeric silsesquioxane (POSS) activation, while the slow release of Ag significantly promotes skin regeneration by reducing inflammation and inhibiting infection [219]. It is important for hydrogel wound excipients to be developed with excellent mechanical strength and tissue adhesion properties. Lu Lu’s team developed a poly(ethylene glycol) diacrylate (PEGDA) covalent network hydrogel [220]. The tensile strength of this composite hydrogel was 81.6 kPa, and the elongation at break was 131.2 ± 10.1%, respectively, which is more than a twofold improvement over PEGEDA hydrogels. Similarly, the composite hydrogel exhibited better ductility. The chitosan composite hydrogels can precisely release drugs and genes. When antimicrobial peptides and plasmid DNA were loaded in the hydrogel, they exhibited sustained release and promoted angiogenesis. Similarly, Chaman Ara et al. crosslinked chitosan/PVA with different concentrations of aminopropyl triethoxy silane (APTES) to produce a hydrogel and investigated its role in angiogenesis [221]. Research shows that the presence of amino group in APTES improves antioxidant properties, and increases the blood flow and oxygen delivery to the wound area to reduce oxidative stress, thus speeding up the healing process. Due to its solubility and 3D structure, PVA provides not only a good texture for hydrogel compositions but also a suitable environment for cell proliferation in damaged areas. Notably, this hydrogel had stronger angiogenic effect. Zhenbing Chen’s group designed and developed a composite chitosan-based hybrid hydrogel, which was synthesized by crosslinking polyethylene glycol star octa-armed polyhedron oligosiloxane (POSS-PEG-CHO) with hydroxypropyl trimethylammonium chloride chitosan (HACC) through Schiff base reaction at the capped end of benzaldehyde [222]. The introduction of POSS can improve the mechanical strength by the large number of crosslinking points in the star-shaped polymer of POSS. This hydrogel regulates inflammation and upregulates the expression of vascular endothelial growth factor (VEGF), promoting vascular and epithelial regeneration. Fewer inflammatory cells and more collagen deposition further promote wound healing and wound tissue regeneration. Xiyun Feng’s team prepared a quaternized chitosan (QCS)/microfibrillated cellulose (MFC) gel from QCS and MFC [223]. The activated partial thromboplastin time (APTT) and prothrombin time (PT) values showed a longer coagulation time. The dense pore walls gave this frozen gel excellent mechanical properties. The QCS/MFC cryogel could maintain 91.2% of the initial maximum stress, demonstrating its high compression fatigue resistance. The QCS/MFC cryogel can come into direct contact with blood and may be used as the tissue-engineered vascular scaffolds.

### 5.3. Chitosan Hydrogels Loaded with Active Ingredients

For the treatment of pro-vascular regeneration, the targeted medication is more effective in providing significant therapeutic benefits. The 3D network structures of chitosan hydrogel coupled with its inherent properties allow it to load with some active ingredients, which can assist in situ treatment, regulate the microenvironment in vivo, promote the regeneration of peripheral blood vessels, and accelerate wound healing [224]. In particular, chitosan hydrogel can retain the load at the defect site, thereby achieving an in situ treatment effect and expediting the treatment process. The combination of bioactive molecules and hydrogel to construct the multifunctional biomaterials is an important and effective mean to promote angiogenesis in tissue engineering.

#### 5.3.1. Pharmaceutical Molecules and Genes

Drugs, gaseous signal molecules, genes, and microRNAs are commonly employed to modulate cell behaviors for the regeneration and tissue engineering, such as cell adhesion, growth, proliferation, and migration. Pharmaceutical molecule and gene therapies offer a promising strategy for promoting angiogenesis and accelerating endothelialization in tissue engineering [225,226,227,228,229,230,231,232,233,234,235,236,237,238,239,240,241,242,243].

Gene therapy-based pro-vascular endothelialization has received considerable attention from researchers, due to the long half-life and high stability of genes compared with growth factors. Conceivably, the integration of plasmids encoding, e.g., VEGF, ZNF580, endothelial nitric oxide synthase (eNOS), etc., possesses ECs activating effects. Hydrogels offer protective and retentive effects, holding immense potential in augmenting the endothelialization of vascular implants. To overcome the inefficiencies associated with gene transfection, we proposed a “dual genes + all-adaptive carrier” idea. Specifically, we innovatively co-delivered both eNOS gene and ZNF580 gene, which collaboratively promote angiogenesis [225]. In order to improve the biocompatibility and gene transfection, our group developed a functional anionic polymer to modify polycationic carriers [226,227,228]. We evaluated the introduction of oligomeric histidine (H-n) sequence into gene vector and found that it promoted endosomal escape and gene transfection and improves the delivery efficiency and expression of pZNF580 [227]. Additionally, a star-shaped functional peptide was synthesized by mixing it with pZNF580 plasmid and then coating it with anionic polymer, which significantly improved gene delivery efficiency and vascularization activity [228,229]. The CAGW peptide and PEG modified amphiphilic copolymer can self-assemble into nanoparticles as a gene carrier, which promotes angiogenesis and endothelialization [230]. We designed the L-arginine-based bioactive carrier to deliver pZNF580 and establish a positive feedback relationship with gene expression with L-arginine within the carrier. The level of ZNF580 (to mediate angiogenesis) and NO (to mediate angiogenesis and anti-inflammation) has been improved. This strategy simultaneously promoted angiogenesis and mitigated inflammation, which co-worked to recover blood flow and rescue limb [231]. Similarly, we have established a “controlled CO release” and “pro-angiogenic gene” dually engineered stimulus-responsive nanoplatform to promote extensive microenvironment normalization and accelerate revascularization [232]. In the treatment of cardiovascular disease, a variety of cells need to be regulated to play a role together. We proposed a combination therapy strategy in which co-delivered DNA and siRNA simultaneously promote ECs proliferation but inhibit SMCs proliferation [233]. Moreover, we also established a CO delivery nanosystem based on regenerative bioactive zinc metal−organic frameworks (MOFs), to normalize the microenvironment by regulating ECs and macrophages (Mφs) through zinc ion and CO release [234]. To alleviate the biotoxicity and instability of therapeutic agents, we co-decorated EC-targeted and mitochondria-localizing-sequence peptides onto Zn-MOF surfaces [235]. We hypothesize the multi-effect drug of endothelium-protective epigenetic inhibitor (JQ1) may preferentially accumulate in injured sites and SMCs to enhance the targeted therapeutic effect. A biomimetic and responsive nanoplatform has been developed for dual-targeted treatment of vascular restenosis [236]. A matrix metalloproteinase (MMP) responsive gene delivery system was constructed to intelligently release genes from the graft surface in situ [237]. Our group reviewed recently developed strategies for improving bioavailability of therapeutic drugs through systemic administration [238]. Additionally, it was confirmed that dual-gene co-delivery and coordinate expression became a “breach” of strengthened gene expression [239]. Double genes coordinate interactions have a significant impact on new blood vessels [240]. We conjugated the REDV-G-TAT-G-NLS-Cys(TP-G) peptide to the amphiphilic copolymer of poly(lactide-*co*-glycolide)-*g*-polyethylenimine (PLGA-*g*-PEI) through isopolyethylene glycol spacer (OPSS-PEG-NHS), and coagglutinated pZNF580 to form the gene complex [241]. Based on Extracellular signal-regulated kinase 2 siRNA (ERK2), a type of ternary delivery system characterized by an endosome-selective-self-accelerating-escape ability was designed and prepared for the purpose of inhibiting the migration of SMCs in vitro [242]. CAGW modified polymer micelles with different hydrophobic cores for efficient gene delivery and for the selective promotion of EC proliferation and migration as well as vascularization [243]. The therapeutic genes, therapeutic gas molecules, and relevant vectors have achieved remarkable results in the field of vascular regeneration and endothelialization.

Chitosan hydrogels have excellent drug loading capacity and controlled release properties. The direct loading of drugs into hydrogels can optimize the desired therapeutic effect of the drugs. Yunen Liu and colleagues reported that Melatonin-loaded hydrogels promote granulation tissue formation and accelerate wound healing by reducing inflammation and promoting angiogenesis and collagen deposition [244]. Melatonin, a hormone produced by the pineal gland in humans and animals, has been shown to have anti-inflammatory effects by regulating the release of inflammatory mediators. Melatonin-loaded hydrogels significantly increased the expression of collagen III, alpha-SMA, and TGF-β1 proteins, accelerating wound healing. In order to induce angiogenesis during bone regeneration, Ihtesham U. Rehman et al. prepared the injectable chitosan/hydroxyapatite/heparin composite hydrogels in situ based on previous studies [150]. Heparin release is expected to occur in a more sustained manner with enhanced blood infiltration and transport due to the increased permeability of layered porous hydrogels. The drug is loaded into the hydrogel, which provides a longer-lasting effect through slow release, while the hydrogel structure protects the activity of drugs and allows the drugs to work properly in the body. With the help of hydrogels, it is possible to achieve in situ therapeutic promotion of vascular regeneration in some areas of tissue engineering, greatly improving the utilization of drugs and the efficiency of drug delivery.

**Figure 7:**
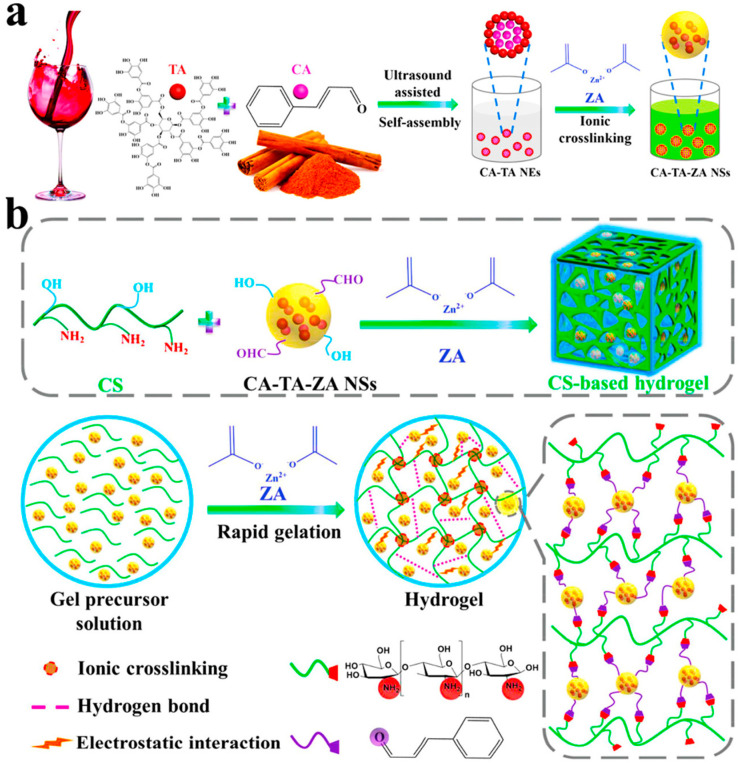
Green regenerative hydrogel wound dressing functionalized by natural drug-food homologous small molecule self-assembled nanospheres. (**a**) The flow chart of the preparation of CA-TA-ZA NSs; (**b**) Synthesis process and potential gelation mechanisms of CS-based hydrogels. Copyright 2022, Wiley [245].

Drugs can be loaded into the hydrogel structure through nanosphere structure to exert therapeutic effects when implanted in the human body. The nanostructure can form a protection layer to prevent the drug from being degraded in the body, and it can also provide a certain retarded release rate. For the first time, an ultrasonic-triggered irreversible tending to equilibrium self-assembly and ionic crosslinking co-driven strategy are proposed: an “integrated” chitosan-based hydrogel network by introducing self-assembled multifunctional nanoparticles, cinnamaldehyde-tannic acid-zinc acetate nanospheres (Figure 7) [245]. The nanoparticle is a combination of hydrophilic and hydrophobic drug-food homologous bioactive small molecules, which has excellent antibacterial and antioxidant properties. These hydrogels achieve rapid in situ gelation at the wound site and completely cover irregular wounds. In addition, it can clean the wound microenvironment, induce skin tissue remodeling, promote blood vessel repair and hair follicle regeneration, then restore the immune system to normal physiological activities, and accelerate wound healing. In summary, this multifunctional chitosan hydrogel shows strong antibacterial, antioxidative, anti-inflammatory, and reducing oxidative stress damage features. This research has developed a drug delivery system modeled on the self-assembly of small molecules prevalent in nature. Chitosan hydrogels have an inherent lack of bioactivity, and strategies for their biofunctional modification are often used to enhance the biofunctional activity of natural polymer hydrogels. The team of Shaobing Zhou prepared an adaptive multifunctional hydrogel with self-healing and injectable properties by making the angiogenic drug deoxyferrioxamine (DFO) into gelatin microspheres and loading them into the composite hydrogel of chitosan and PVA quaternary ammonium of 3-carboxy-4-fluorobenborate [246]. The multifunctional hydrogel can not only reduce ROS but can also release DFO in response to matrix metalloproteinases. Chuanglong He et al. developed an in situ crosslinked aldehyde hyaluronic acid (AHA)/N, O-carboxymethyl chitosan (NOCC) nanocomposite hydrogel and modified mesoporous silica nanoparticles (MSNs) with a polyelectrolyte loaded with the small molecule drug of 1-phosphosphingosin (S1P) [247]. The nanocomposite hydrogel effectively controls the release of S1P to the defect area and significantly induces the growth of neovasculum to the tissue engineering scaffold. Jun Wu et al. developed the chitosan methacrylate gallic acid (CSMA-GA) hydrogels to encapsulate drugs in a complex hydrogel [248]. The composite hydrogel promotes angiogenesis by upwarding the levels of VEGF and platelet endothelial cell adhesion molecule-1 (CD31), which can achieve rapid wound healing. Responsive hydrogels can release the loaded drugs to regulate the microenvironment. The diffusion rate of drugs is difficult to control in vivo, but, with the help of nanoparticles, the drug release and local delivery are possible, in which the chitosan hydrogel provides a suitable delivery vehicle to maintain the activity of biological factors.

#### 5.3.2. Stem Cells

Hydrogels are superior functional cell delivery vehicles as they effectively protect cells from external oxidative stress, ensure the biological activity of stem cells, and allow for the continuous release of cells so that they can perform their intended function. Stem cells have excellent advantages as biologically active substances, secreting a wide range of angiogenic growth factors and having a high angiogenic potential, which helps to accelerate the remodeling process of damaged tissue. Stem cells play an important role in promoting the development of tissue engineering and regenerative medicine. Mesenchymal stem cells (MSCs) have the potential of self-renewal and multidirectional differentiation, which make them a promising avenue for treating diseases and injuries. The biomaterials loaded with stem cells show structural similarity to the natural matrix components, thereby serving as an excellent strategy for enhancing the pro-angiogenic activity of MSCs in a clinical setting. Zongjin Li et al. worked on the co-transplantation of nitric oxide (NO)-releasing chitosan (CS-NO) hydrogel with human placenta-derived MSC (hP-MSCs) [249]. They verified that NO hydrogel significantly improved the survival rate of hP-MSCs transplanted, and the CS-NO and hp-MSCs combined hydrogel improved the functional recovery of the ischemic hindlimb. Similarly, adipose-derived stem cells (ASCs) secrete a variety of angiogenic growth factors and are thus being investigated as a potential therapy for ischemic tissue. They have great potential for therapeutic angiogenesis and clinical application. Tai-Horng Young and colleagues developed a thermosensitive chitosan/gelatin hydrogel [250]. The addition of gelatin in the hydrogel significantly improved the activity and stability of encapsulated ASCs, and the composite hydrogel was able to release ASCs slowly. The hydrogel showed good tubule formation potential and significantly increased capillary density. Santanu Dharaa et al. proposed a crosslinked acellular bone ECM (DBM) and fatty acid-modified chitosan (oleyl chitosan, OC)-based biohybrid hydrogel (DBM/OC) loaded with human amniotic stem cells (HAMSCs) [251]. The interaction between stem cells and ECM may improve site-specific tissue remodeling. DBM/OC hydrogels have good potential of neovasculature and have potential as substrates for tissue engineering scaffolds. Shan-hui Hsu et al. encapsulated neural stem cells (NSCs) and ECs cospheres into chitosan or gelatin based hydrogels to support the long-term growth of cell globules in a 3D environment [252]. Compared with ECs on chitosan substrate, NSC and ECs co-cultured on hyaluronan-grafted chitosan (CS-HA) rapidly assemble into larger and more compact coglobules. Moreover, ECs embedded in NSC/EC cospheres in gelatine-based hydrogels containing fibroblast growth factor 2 (FGF2) have greater cladding-forming potential, as indicated by the formation of tubular structures from the surface of cospheres after induction of FGF2. Zongjin Li et al. prepared a hydrogel with insulin-like growth factor-1 [253]. By improving the retention rate of transplanted cells and by promoting angiogenesis and blood flow recovery, the generation of collateral vessels at the ischemic site was significantly improved; the formation of neovascularization was enhanced; and the limb rescue was improved.

#### 5.3.3. Stem Cell Exosomes

**Figure 8:**
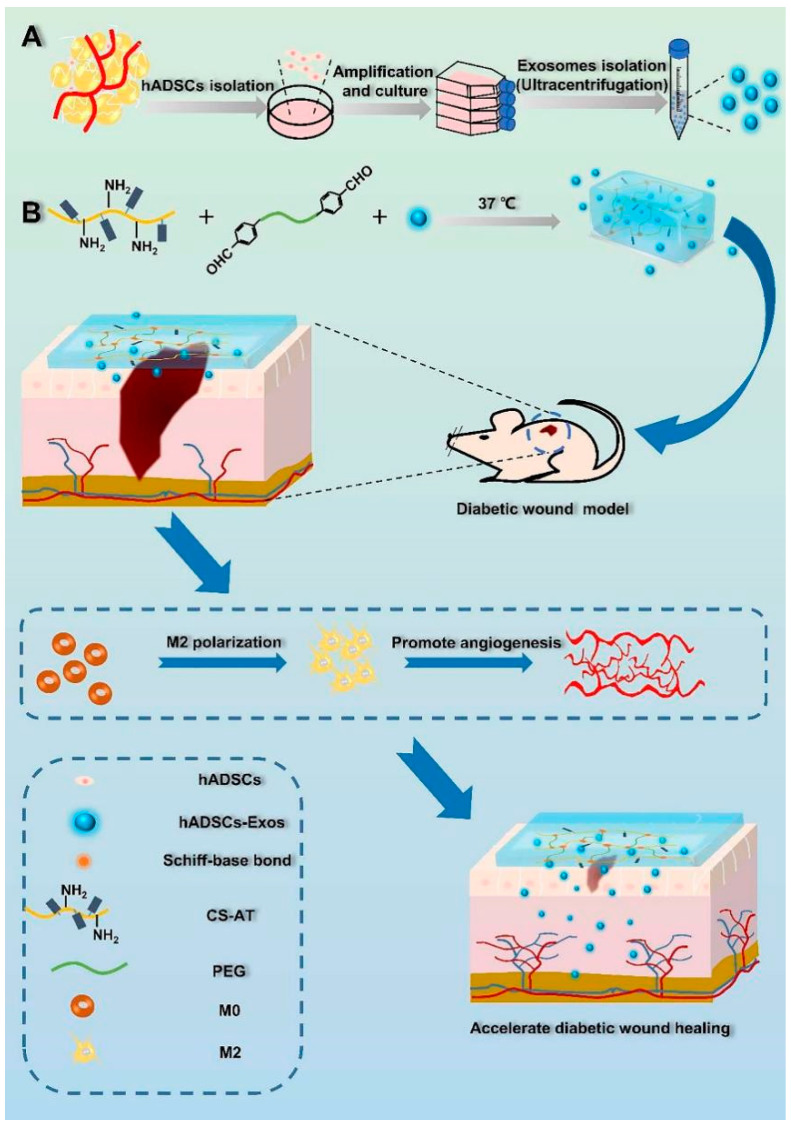
Schematic description revealing the fabrication of (**A**) hADSCs-Exos, (**B**) CS-AT-Exo hydrogel, and the larvaceous application in the diabetic wound healing process. Exosomes laden self-healing injectable hydrogel enhances diabetic wound healing via regulating macrophage polarization to accelerate angiogenesis. Copyright 2022, Elsevier [256].

As the key to cell-free therapeutic strategies, exosomes reduce the occurrence of immune reactions that can be induced during stem cell transplantation, can effectively reduce safety issues in humans, play a key role in disease treatment, and have been a hot spot for tissue engineering research in recent years. However, their poor storage and low stability make it difficult to use them directly in tissue engineering medical applications. Hydrogels play a role in cell-free therapeutic strategies. Chitosan hydrogels can retain cells or slow-release drugs in situ for longer periods of time and have the same loading and slow-release effect on exosomes, which remains a good carrier basis for tissue-engineered vascular regeneration treatments. As mentioned above, the tunable biological and physicochemical properties of chitosan hydrogels provide the basis for this therapeutic strategy. Hydrogels loaded with stem cells can promote wound healing; however, the potential risk of delivering stem cells or forming tumors or ectopic tissue is also a concern due to the delivery method. Stem cell-derived exosomes are considered to be the main secretory product of stem cells. Nanoscale exosomes are more readily endocytosed by cells; then, they transfer efficient mRNA, miRNA, and proteins into target cells. Stem cell exosomes are enriched with over 100 extracellular matrix proteins, messenger RNAs, and multiple growth factors, which can facilitate the regenerative process while avoiding or mitigating the risk of rejection and inflammation in tissue engineering. A self-healing hydrogel was prepared from hydroxybutylchitosan and konjac glucomannan oxide through a reversible Schiff base reaction [254]. This hydrogel was loaded with exosomes derived from bone mesenchymal stem cells, which reduced the leakage of exosomes after the destruction of the hydrogel network, thus showing great potential in promoting angiogenesis, tissue reconstruction, and regeneration. Zongjin Li and colleagues combined hP-MSC-derived exosomes with chitosan hydrogels, which helped to protect exosomes from being cleared by the host immune system through the immune isolation barrier function of shell hydrogels [255]. The results showed that the chitosan hydrogel significantly improved the stability of proteins and microRNAs in exosomes, as well as enhanced the retention of exosomes in vivo. The exosome and hydrogel treatment significantly promoted endogenous angiogenesis in ischemic tissue, which played roles in muscle protection. Chenggang Yi et al. developed a self-healing exosome-loaded injectable hydrogel based on chitosan grafted aniline tetamer (CS-AT) and dibenzaldehyde polyethylene glycol (PEG-DA) to solve the problem of relatively short half-life and rapid elimination of exosomes in vivo. [256]. The hydrogel enhanced the immune regulation of macrophages, promoted the differentiation of macrophages toward M2 phenotype, and was conducive to vascular regeneration (Figure 8). In bone tissue engineering, microvascular networks are essential for the survival needs of cells that complement angiogenesis and osteogenesis and promote bone regeneration. Xiaojuan Wei et al. reported a thermosensitive hydrogel formed by etopochitosan/β-glycerophosphate with bone mesenchymal stem cell-derived small extracellular vesicles (BSMC-sEVs) [257]. The hydrogel has a good sustained release of BSMC-sEVs and can dose-dependently up-regulate the expression of osteogenic genes, increase tube formation, and promote angiogenesis.

#### 5.3.4. Proteins

The vascular regeneration is a complex therapeutic process in which a single biomaterial cannot meet the needs for bioactivity. Composite hydrogels loaded with bioactive components have become a favorite for this field because of their multiple properties. Protein-like substances have prominent pro-vascularization effects, such as vascular endothelial growth factors and other cytokines, but they have a short half-life in vivo, limiting their application. Fortunately, chitosan hydrogels show excellent protective properties for therapeutic protein agents. With the help of chitosan hydrogels, they can be protected from decomposition, thereby enhancing their potential therapeutic benefits.

**Figure 9:**
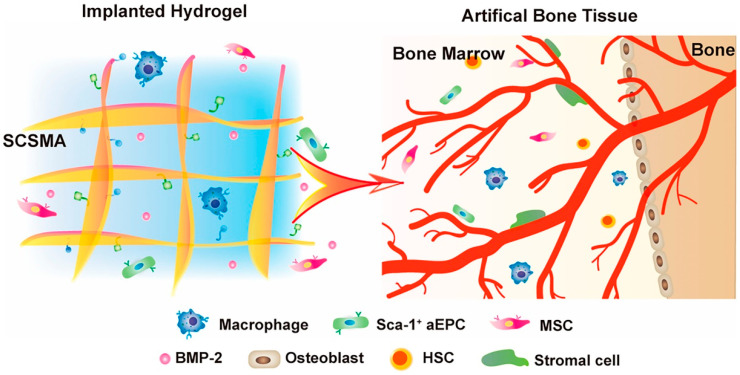
Effect of sulfated chitosan hydrogel on vascularization and osteogenesis. Copyright 2022, Elsevier [258].

Skin defect repair requires a complex interplay of cellular processes such as cell adhesion, diffusion, migration, and angiogenesis. Owing to its structure, sulfate chitosan shows the similar biological growth factor effect as heparin. Jing Wang et al. designed and synthesized methacrylated sulfated chitosan (SCSMA) hydrogels through the violet linkage system. They loaded bone morphogenetic protein-2 (BMP-2) into the hydrogel [258]. This BMP-2-/SCSMA hydrogel can create an immune microenvironment with successful vascularization effect (Figure 9). Ju Zhang and colleagues identified LL-37 peptide, a member of the human antimicrobial peptide family, which contributes to the formation of blood vessels and is widely used in wound healing [259]. They prepared LL-37/chitosan hydrogel by physical blending method. The LL-37/CS hydrogel increased the protein expression of hypoxia inducible factor-1α, transforming growth factor-β and vascular endothelial growth factor-A, then increased blood supply, promoted the generation of granulation tissue, and even protected tissue matrix catabolism. This hydrogel can promote wound healing and improve keratinocyte re-epithelialization, which may play a certain reference significance for chronic inflammation and remodeling vascular microcirculation. Substance P (SP) is a kind of neuropeptide, which has a variety of physiological functions for cells. It is easy to be degraded by angiotensin converting enzyme and matrix metalloproteinase in vivo. To address this issue, Changqing Zhang et al. prepared a stable SP-conjugated chitosan hydrochloride hydrogel for in vitro administration [260]. This hydrogel can promote the proliferation, migration, tube formation, and expression of genes and proteins related to angiogenesis of ECs. Some amino acids support ECs proliferation and trigger angiogenesis during wound healing. Muhammad Yar’s team loaded three structurally closed amino acids (arginine, alanine, and phenylalanine) separately into the chitosan/collagen hydrogel [261]. The study found that the incorporation of arginine into the hydrogel resulted in a significantly high level of angiogenesis stimulation, which presents promising potential for skin tissue engineering applications of arginine-based hydrogel. Loaded with proteins and free of exogenous cells or cytokines, chitosan hydrogel regulates cellular behavior in the body and promotes normalization of the microenvironment, thereby accelerating vascular regeneration and wound healing. In some studies, the proteases loaded form a cascade reaction in vivo, allowing the therapeutic agents to work more efficiently. The supply of oxygen can effectively promote the reparative processes of angiogenesis. Zhiguo Li et al. designed a multifunctional glucose oxidase (GOx) and catalase (CAT) nanase-chitosan (GCNC) hydrogel complex [262]. The hydrogel complex has cascaded catalytic and self-enhancing antibacterial properties, providing continuous oxygen delivery for efficient wound healing. Moreover, it stimulates cellular proliferation, migration, and angiogenesis. At the same time, gluconic acid, a by-product of the cascade reaction, activates the amino group of chitosan and enhances its antimicrobial properties. Changsheng Liu’s team explored the strategies to regulate macrophage behaviors with a sulfated chitosan-doped collagen type I hydrogel in pathological microenvironments [263]. The macrophages participate in the proliferative and remodeling phases of wound healing by differentiating into fibroblasts. The manipulation of the wound inflammation microenvironment by regulating macrophage behavior has attracted much attention in recent years [232,234].

#### 5.3.5. Bioactive Glass

Bioactive glass is a kind of “bioactive materials” for bone defect repairing, which possesses a remarkable ability to bond with living tissue. The most striking feature of bioactive glass is its ability to bind to living tissue [264]. Studies have found that wound dressings containing boric acid bioactive glass can induce angiogenesis and repair full-thickness wound in animal models. This makes bioactive glass a promising therapeutic candidate for the tissue engineering of vascularized tissues [264]. Injectable thermosensitive hydrogel has rapid and reversible sol-gel transformation behavior and is easy to fill irregular wound defects. However, its weak tissue adhesion restricts its clinical application.

To solve this problem, Xiaoli Zhao et al. established a clinical treatment technique for complex wounds [265]. They loaded the nanobioactive glass (80 SiO_2_, 16 CaO, and 4 P_2_O_5_ in mol%) into hydrogel. The active ions released from the hydrogel can up-regulate the gene expression of VEGF and b-FGF, stimulate EC migration and angiogenesis. Peiman Brouki Milan et al. prepared the copper-doped borate bioactive glass and thiol chitosan/oxidized carboxymethyl cellulose as a dressing material to accelerate wound healing [10]. This hydrogel containing boric acid bioactive glass has sufficient potential to occlude wounds and improve blood vessel formation throughout the wound repair process in vivo. Xin Wu and Haiyan Li added bioactive glass to alginate/carboxymethyl chitosan hydrogel to obtain bioactive hydrogel [266]. The active ions and hydrogel can stimulate RAW 264.7 cells to polarize to M2 extremes, enhance the paracrine interaction between activated macrophages and repair cells, stimulate the migration of repair cells, and promote angiogenesis and extracellular matrix deposition.

In this part, researchers developed chitosan hydrogel scaffolds loaded with bioactive glass, which benefit from the special composition and silicone network structure of bioactive glass and the release of Si, Ca, and P elemental components (in the form of ions or ionic groups) to regulate cell behavior, such as the directional differentiation of stem cells and the phenotypic polarization of macrophages, thus exerting an anti-inflammatory, pro-angiogenic effect. This has led researchers to focus on whether some of these ions in humans have therapeutic effects. Chitosan hydrogels also provide the structural basis for the release of ions, enabling a stable and long-lasting therapeutic process. Bioactive glasses also enhance the biocompatibility and angiogenic activity of the hydrogel, presenting potential for development in the field of bone repair and providing some insight and inspiration for the proposal and study of ion therapy strategies in other tissue engineering fields.

#### 5.3.6. Others

Inspired by hot springs, Jiang Chang and collaborators designed and developed a ferromagnesium/N, O-carboxymethyl chitosan (FA-NOCs) photothermal bioactive composite hydrogel [267]. In this work, the photothermal property and ion release of ferromagnesite ceramic powder and composite hydrogel demonstrated a strong synergistic effect in activating different angiogenic factors and signaling pathways to promote angiogenesis. This study confirmed that the combination of bioactive ions and mild heat treatment may be an effective way to enhance tissue regeneration with broad research prospects. Hui Zeng and collaborators prepared a Mg-containing double-crosslinked hydrogel by introducing active Mg^2+^ into the system via the coordination bonds of MgS [268]. The introduction of POSS and Mg^2+^ into the composite hydrogel effectively promoted cell adhesion and angiogenesis. Similarly, this team added MgO nanoparticles to a newly synthesized aqueous solution of water-soluble creatine phosphate functionalized chitosan to produce the corresponding hydrogel, which promoted osteogenic differentiation of MC3T3-E1 cell and the tube formation of HUVECs [269]. This suggests that Mg^2+^ and MgO have a therapeutic effect on vascular regeneration, which provides ideas for the redevelopment and innovation of therapeutic drugs and expands the therapeutic field of tissue engineering.

**Figure 10:**
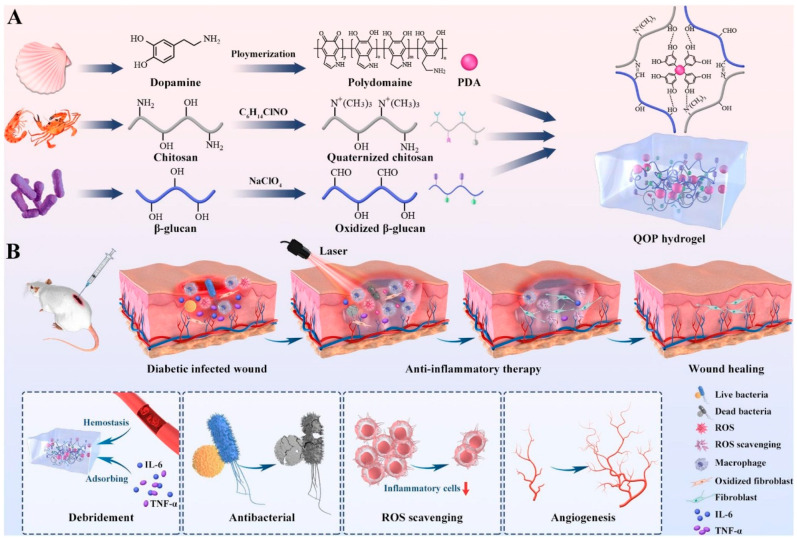
Illustration of the fabrication of multifunctional QOP hydrogel for treating bacterially infected diabetic wounds. (**A**) The formation of QOP hydrogel is achieved through the Schiff base reaction between the aldehydes of oxidized β-glucan and the hydrazides of quaternized chitosan. (**B**) Using a simply mixed injection, the QOP hydrogel dressing is quickly constructed to tightly cover diabetic wounds, orderly promoting wound healing by adsorbing exudates, reducing inflammation, and guiding tissue regeneration. All-in-one: harnessing multifunctional injectable natural hydrogels for ordered therapy of bacteria-infected diabetic wounds. Copyright 2022, Elsevier [270].

Studies have shown that the doping of dopamine nanoparticles can improve the biological and mechanical properties of chitosan hydrogels to a certain extent. To this end, Jianliang Shen’s team developed an in situ injectable hydrogel (QOP) composed of a polysaccharide matrix (chitosan quaternary ammonium salt and oxidized β-glucan) and polydopamine nanoparticles (PDA NPs) [270]. The QOP hydrogel can reduce inflammation, accelerate granulation tissue formation and collagen deposition, promote neovascularization, and reshape the microenvironment in vivo (Figure 10). Dopamine nanoparticles improve the biological and mechanical properties of chitosan hydrogels, while exhibiting certain bioactive functions.

The augmentation of wound healing through the creation of oxygen-rich microenvironments presents a promising avenue of exploration. In a recent study, Fatemeh Dadkhah Tehrani et al. reported an oxygen-producing material based on chitosan/β-glycerophosphate (β-GP) thermosensitive hydrogel [271]. They encapsulated the hydrogen peroxide in poly(lactic acid) particles, which were then embedded in a heat-sensitive hydrogel. The administration of this compound atop an amniotic membrane sheet covering the wound has the potential to supply both oxygen and the biological factors, leading to a synergistic effect and ultimately promoting wound healing. The ultimate goal of oxygen therapy is to enhance wound healing. Chitosan hydrogels are permeable to oxygen and water, loaded with peroxides that act as a source of oxygen in the body, and they also provide a protective layer for hydrogen peroxide, avoiding the explosive release of oxygen due to oxidative stress in the microenvironment. This provides a new breakthrough point for wound healing by oxygen treatment using chitosan composite hydrogels.

To meet practical requirements, chitosan hydrogels have been adjusted to the mechanical structure, biological properties, and physicochemical properties of the gels from their own raw materials and scaffold manufacturing processes. The selection and adjustment of the crosslinking agent and crosslinking method allows the chitosan hydrogel to be structurally adapted and optimized. Chitosan hydrogels and production processes are adapted to each other and contribute to each other. The combination of natural materials with chitosan hydrogels enhances biocompatibility and biofunctionality. To enhance the mechanical properties of chitosan-based hydrogels to make them more suitable for tissue engineering applications, numerous artificial materials have been used to compound with chitosan hydrogels, with promising improvements in biological functionality. As for the biotherapeutic activity of chitosan hydrogels applied to vascular regeneration, the composite hydrogel significantly enhances the therapeutic ability to target pro-vascular regeneration, usually by taking advantage of its good loading and slow-release properties and loading the relevant therapeutic drugs and cells. In summary, chitosan and its modified hydrogels have a variety of applications in the field of vascular regeneration. The overall summary is shown in Table 3.

## 6. Future Perspectives

As a kind of natural polymer material, chitosan is abundant in nature, which has good compatibility and can promote the interaction between tissues and cells. The rich active functional groups enable chitosan with a variety of properties for wider applications. The chitosan hydrogels are often used in regenerative medicine and tissue engineering owing to the advantages of their three-dimensional porous extracellular matrix-like structure, high water content which could provide a moist microenvironment, the ability to fill irregularly shaped wounds and tissues, and good biocompatibility and blood compatibility [272,273]. The chitosan hydrogels can not only provide a moist healing environment for local damaged tissues but also serve as a biomimic matrix for cell growth meanwhile controlling the release of drugs, peptides, proteins, genes, bioactive glass, and other bioactive substances [274,275]. In view of the shortcomings of hydrogels in terms of mechanical properties, a lot of research efforts have been invested in the hope of compensating and improving them [276,277]. It is a feasible idea and strategy to enhance the mechanical properties through structural design, and the technical breakthrough in this aspect will be a research hotspot in the future.

Chitosan hydrogels play an important role in vascular remodeling and regeneration via drug and growth factor release. For example, chitosan hydrogels provide a stem cell niche by protecting stem cells from host immune cells [278]. The composite hydrogels inoculated with stem/stromal cells can enhance tissue remodeling and repair by providing cells and growth factors/cytokines at the defect site for a long period of time [279,280]. With the continuous development of science and technology, the single-function biological materials are not enough to meet the actual clinical needs. The development of multifunctional hydrogels is imperative for tissue engineering and regenerative medicine. The current researches mostly use chitosan hydrogels as slow-release carriers loaded with various active substances to create an in vivo environment for tissue regeneration through in situ therapy or targeted therapy, such as supplying oxygen [262], promoting angiogenesis [194,257,281], reducing ROS stimulation [282], etc. The combination of chitosan hydrogels with emerging techniques for the preparation of tissue engineering scaffolds has also created a stronger research dynamic. The 3D printing technology offers hope for individualization of tissue engineering, while the mechanical properties of chitosan hydrogels can be improved to suit more clinical needs with the help of technological innovations [283,284]. Innovations in the preparation technology and optimization of hydrogels will complement each other to promote the progress of tissue engineering and regenerative medicine.

## Figures and Tables

**Table 1:** Other common techniques used to make tissue engineering scaffolds and their features.

Technology	Methods/Principle	Advantages	Disadvantages	References
Biological coating technology	Plasma spraying Chemical deposition Biomimetic mineralization Laser cladding Electrochemical deposition layer-by-layer self-assembly; .etc	High repeatability; Controlled process; Simple process; High deposition efficiency.	Unstable coating uniformity; Low bond strength.	[53,58,61]
Electrospinning technology	Electrostatic atomization of polymeric fluids for jet spinning in a strong electric field	For the repair of tissues and organs; Small pore size; High porosity; Good fibre homogeneity.	Fibre structure regulation; Low spinning efficiency; Limited variety of raw materials.	[70,76,80]
3D printing technology	Based on digital model files Using bondable materials Constructs objects by printing layer by layer	Save material; High precision; High level of complexity; Distributed production; Low cost.	Difficult to produce on a large scale; Limited variety of materials; Equipment maintenance problems.	[83,84]
Injectable scaffolds	Formation of hydrogel materials by physical/chemical crosslinking	Good biocompatibility; Protects cells; Easy to modify.	Poor mechanical properties; Difficult to disinfect.	[87,90,92,96]
Particle to pore	Solution pouring/particle leaching	Pore size/porosity can be adjusted independently; Wide applicability; Modifiable solid core surface.	The scaffold is brittle; Pore-forming agents tend to remain/agglomerate; Organic solvents are often used; Inability to apply soft tissue.	[104,105,106]
Phase separation/Freeze drying method	Emulsion freeze-drying Solution freeze-drying Hydrogel freeze-drying	The scaffold is elastic; The penetration of the hole is good; Easy to introduce active substances; Achieving controlled microarchitectures; Easy to produce industrially.	Not easy preparation of large diameter stents; Avoid high temperature; Large pore size.	[107,108,109,110]
Gas foaming	Physical foaming method Chemical foaming method	No organic solvents are used (Physical); The reaction temperature is low (Physical); High porosity (Chemical); Good connectivity (Chemical).	Uncontrolled porosity and pore size (Physical); Low connectivity rate (Physical); Blowing agent requirements are high (Chemical); Closed porous structure.	[111,112]
Microspheres aggregation	Through heat treatment, the contact of the microspheres is linked together by chain motion	Good hole connection; Aperture easy to control; Controlled release; Regulation of drug release rate.	Small aperture; Low porosity.	[113]

**Table 3:** Chitosan hydrogels and their applications in vascular remodeling.

Type	Technology	Combining Materials		References
Chitosan and its derivative hydrogels	Physical and chemical crosslinking methods	-		[187,188,189,190,191]
	Domain modification	-		[192,193,194]
	3D printing	-		[195,196,197,198,199,200]
	Electrospinning and freeze-drying methods	Silk fibroin fiber; heparinized thermoplastic polyurethane co-electrospun fiber		[201]
Chitosan composite hydrogels	-	Natural materials	β-glycerophosphates (β-GP)	[208]
			Fibrin	[207,209]
			κ-carrageenan	[211]
			Sulfonated sodium alginate	[212]
	-	Artificial materials	Polyvinyl alcohol (PVA)	[187]
			Polydopamine modified carbon nanotubes	[214]
			Hyaluronate-dopamine	[210]
			Gelatin-methacryloyl (GelMA); 3-buten-1-amine (BA)	[215]
			Poly-hedral oligomeric silsesquioxane (POSS)	[219]
			Poly(ethylene glycol) diacrylate (PEGDA)	[220]
			Aminopropyl triethoxy silane (APTES)	[221]
			Polyeth-ylene glycol star octa-armed polyhedron oligosiloxane (POSS-PEG-CHO)	[222]
			microfibrillated cellulose (MFC)	[223]
Chitosan hydrogels loaded with active ingredients	-	Genes	endothelial nitric oxide synthase(eNOS)	[225]
			ZNF580	[225,226,227,228]
			Matrix metalloproteinase (MMP)	[237]
	-	Pharmaceutical molecules	CO	[234]
			Thyroxine	[244]
			Heparin	[150]
			Cinnamaldehyde-tannic acid-zinc ace-tate nanospheres	[245]
			Deoxyferriox-amine (DFO)	[246]
			1-phosphosphingosin (S1P)	[247]
			Amphiphilic Pluronic F127 molecules	[248]
	-	Stem cells	Mesenchymal stem cells (MSCs)	[253]
			Human placenta derived MSC (hP-MSCs)	[249]
			Adipose-derived stem cells (ASCs)	[250]
			Human amniotic stem cells (HAM-SCs)	[251]
			Neural stem cells (NSCs)	[252]
	-	Stem cell exosomes (derived from)	bone mesenchymal stem cells	[254,257]
			Human placenta derived MSC (hP-MSCs)	[255]
			Human adipose-derived mesenchymal stem cells	[256]
	-	Proteins	Bone morphogenetic protein-2 (BMP-2)	[258]
			LL-37 peptide	[259]
			Substance P (SP)	[260]
			Arginine	[261]
			Glucose oxidase (GOx)	[262]
			Collagen type I	[263]
	-	Bioactive glass		[264,265,266]
	-	Others	Ferromagnesium	[267]
			Mg^2+^	[268,269]
			Polydopamine nanoparticles (PDA NPs)	[270]
			Hydrogen peroxide (H_2_O_2_)	[271]

## Data Availability

Data will be made available on request.

## References

[1] Tavakol D.N., Fleischer S., Falcucci T., Graney P.L., Halligan S.P., Kaplan D.L., Vunjak-Novakovic G. (2021). Emerging Trajectories for Next Generation Tissue Engineers. Acs Biomater. Sci. Eng..

[2] Jenndahl L., Osterberg K., Bogestal Y., Simsa R., Gustafsson-Hedberg T., Stenlund P., Petronis S., Krona A., Fogelstrand P., Strehl R. (2022). Personalized tissue-engineered arteries as vascular graft transplants: A safety study in sheep. Regen. Ther..

[3] Gao Y., Li Z., Huang J., Zhao M., Wu J. (2020). In situformation of injectable hydrogels for chronic wound healing. J. Mater. Chem. B.

[4] Xu J., Feng Q., Lin S., Yuan W., Li R., Li J., Wei K., Chen X., Zhang K., Yang Y. (2019). Injectable stem cell-laden supramolecular hydrogels enhance in situ osteochondral regeneration via the sustained co-delivery of hydrophilic and hydrophobic chondrogenic molecules. Biomaterials.

[5] Stefani R.M., Lee A.J., Tan A.R., Halder S.S., Hu Y., Guo X.E., Stoker A.M., Ateshian G.A., Marra K.G., Cook J.L. (2020). Sustained low-dose dexamethasone delivery via a PLGA microsphere-embedded agarose implant for enhanced osteochondral repair. Acta Biomater..

[6] Islam M.M., Shahruzzaman M., Biswas S., Sakib M.N., Rashid T.U. (2020). Chitosan based bioactive materials in tissue engineering applications-A review. Bioact. Mater..

[7] Ways T.M.M., Lau W.M., Khutoryanskiy V.V. (2018). Chitosan and Its Derivatives for Application in Mucoadhesive Drug Delivery Systems. Polymers.

[8] Zhou S., Bei Z., Wei J., Yan X., Wen H., Cao Y., Li H. (2022). Mussel-inspired injectable chitosan hydrogel modified with catechol for cell adhesion and cartilage defect repair. J. Mater. Chem. B.

[9] Zhang J., Gao Z., Zhang Y., Tian J. (2020). Study on Chitosan-Based Nanocomposite Hydrogel in Soft Tissue Defect of Hand. Nanosci. Nanotechnol. Lett..

[10] Mehrabi A., Karimi A., Mashayekhan S., Samadikuchaksaraei A., Milan P.B. (2022). In-situ forming hydrogel based on thiolated chitosan/carboxymethyl cellulose (CMC) containing borate bioactive glass for wound healing. Int. J. Biol. Macromol..

[11] Yin S., Zhang W., Zhang Z., Jiang X. (2019). Recent Advances in Scaffold Design and Material for Vascularized Tissue-Engineered Bone Regeneration. Adv. Healthc. Mater..

[12] Zhang J., Tong D., Song H., Ruan R., Sun Y., Lin Y., Wang J., Hou L., Dai J., Ding J. (2022). Osteoimmunity-Regulating Biomimetically Hierarchical Scaffold for Augmented Bone Regeneration. Adv. Mater..

[13] Wang X., Yu Y., Yang C., Shao C., Shi K., Shang L., Ye F., Zhao Y. (2021). Microfluidic 3D Printing Responsive Scaffolds with Biomimetic Enrichment Channels for Bone Regeneration. Adv. Funct. Mater..

[14] Huling J., Min S.-i., Kim D.S., Ko I.K., Atala A., Yoo J.J. (2019). Kidney regeneration with biomimetic vascular scaffolds based on vascular corrosion casts. Acta Biomater..

[15] Duijvelshoff R., Cabrera M.S., Sanders B., Dekker S., Smits A.I.P.M., Baaijens F.P.T., Bouten C.V.C. (2020). Transcatheter-Delivered Expandable Bioresorbable Polymeric Graft With Stenting Capacity Induces Vascular Regeneration. JACC-Basic Transl. Sci..

[16] Zhao L., Li X., Yang L., Sun L., Mu S., Zong H., Li Q., Wang F., Song S., Yang C. (2021). Evaluation of remodeling and regeneration of electrospun PCL/fibrin vascular grafts in vivo. Mater. Sci. Eng. C-Mater. Biol. Appl..

[17] Liu J., Qin Y., Wu Y., Sun Z., Li B., Jing H., Zhang C., Li C., Leng X., Wang Z. (2019). The surrounding tissue contributes to smooth muscle cells’ regeneration and vascularization of small diameter vascular grafts. Biomater. Sci..

[18] Peifen M., Mengyun L., Jinglong H., Danqian L., Yan T., Liwei X., Han Z., Jianlong D., Lingyan L., Guanghui Z. (2022). New skin tissue engineering scaffold with sulfated silk fibroin/chitosan/hydroxyapatite and its application. Biochem. Biophys. Res. Commun..

[19] Janmohammadi M., Nourbakhsh M.S., Bonakdar S. (2021). Electrospun Skin Tissue Engineering Scaffolds Based on Polycaprolactone/Hyaluronic Acid/L-ascorbic Acid. Fibers Polym..

[20] Altun E., Aydogdu M.O., Togay S.O., Sengil A.Z., Ekren N., Haskoylu M.E., Oner E.T., Altuncu N.A., Ozturk G., Crabbe-Mann M. (2019). Bioinspired scaffold induced regeneration of neural tissue. Eur. Polym. J..

[21] Niu Y., Galluzzi M. (2020). A biodegradable block polyurethane nerve-guidance scaffold enhancing rapid vascularization and promoting reconstruction of transected sciatic nerve in Sprague-Dawley rats. J. Mater. Chem. B.

[22] Zhao B., Zhao Z., Ma J., Ma X. (2019). Modulation of angiogenic potential of tissue-engineered peripheral nerve by covalent incorporation of heparin and loading with vascular endothelial growth factor. Neurosci. Lett..

[23] Fernandez-Perez J., Kador K.E., Lynch A.P., Ahearne M. (2020). Characterization of extracellular matrix modified poly(epsilon-caprolactone) electrospun scaffolds with differing fiber orientations for corneal stroma regeneration. Mater. Sci. Eng. C-Mater. Biol. Appl..

[24] Kim D.K., Lee S., Choi J.H., Jung B.S., Kim K.S., Song J.E., Reis R.L., Khang G. (2021). Enhanced Silk Fibroin-Based Film Scaffold Using Curcumin for Corneal Endothelial Cell Regeneration. Macromol. Res..

[25] Xue Y., Kim H.-J., Lee J., Liu Y., Hoffman T., Chen Y., Zhou X., Sun W., Zhang S., Cho H.-J. (2022). Co-Electrospun Silk Fibroin and Gelatin Methacryloyl Sheet Seeded with Mesenchymal Stem Cells for Tendon Regeneration. Small.

[26] Wang H., Wu Y., Yao Z., Wang C. (2020). Study of a new nano-hydroxyapatite/basic fibroblast growth factor composite promoting periodontal tissue regeneration. Mater. Express.

[27] Sahbazoglu K.B., Demirbilek M., Bayari S.H., Buber E., Toklucu S., Turk M., Karabulut E., Akalin F.A. (2022). In vitro comparison of nanofibrillar and macroporous-spongious composite tissue scaffolds for periodontal tissue engineering. Connect. Tissue Res..

[28] Yao Y., Raymond J.E., Kauffmann F., Maekawa S., Sugai J.V., Lahann J., Giannobile W.V. (2022). Multicompartmental Scaffolds for Coordinated Periodontal Tissue Engineering. J. Dent. Res..

[29] Thottappillil N., Nair P.D. (2020). Dual source co-electrospun tubular scaffold generated from gelatin-vinyl acetate and poly-epsilon-caprolactone for smooth muscle cell mediated blood vessel engineering. Mater. Sci. Eng. C Mater. Biol. Appl..

[30] Dastagir K., Dastagir N., Limbourg A., Reimers K., Strauss S., Vogt P.M. (2020). In vitro construction of artificial blood vessels using spider silk as a supporting matrix. J. Mech. Behav. Biomed. Mater..

[31] Altinova H., Hammes S., Palm M., Achenbach P., Gerardo-Nava J., Deumens R., Fuehrmann T., van Neerven S.G.A., Hermans E., Weis J. (2020). Dense fibroadhesive scarring and poor blood vessel-maturation hamper the integration of implanted collagen scaffolds in an experimental model of spinal cord injury. Biomed. Mater..

[32] Limongi T., Brigo L., Tirinato L., Pagliari F., Gandin A., Contessotto P., Giugni A., Brusatin G. (2021). Three-dimensionally two-photon lithography realized vascular grafts. Biomed. Mater..

[33] Chandel A.K.S., Shimizu A., Hasegawa K., Ito T. (2021). Advancement of Biomaterial-Based Postoperative Adhesion Barriers. Macromol. Biosci..

[34] Wang L., Cao Y., Shen Z., Li M., Zhang W., Liu Y., Zhang Y., Duan J., Ma Z., Sang S. (2022). 3D printed GelMA/carboxymethyl chitosan composite scaffolds for vasculogenesis. Int. J. Polym. Mater. Polym. Biomater..

[35] Ichanti H., Sladic S., Kalies S., Haverich A., Andree B., Hilfiker A. (2020). Characterization of Tissue Engineered Endothelial Cell Networks in Composite Collagen-Agarose Hydrogels. Gels.

[36] Li L., Liu W., Zhao Y., Ma P., Zha S., Chen P., Lu H., Jiang X., Wan S., Luo J. (2020). Dual-Peptide-Functionalized Nanofibrous Scaffolds Recruit Host Endothelial Progenitor Cells for Vasculogenesis to Repair Calvarial Defects. Acs Appl. Mater. Interfaces.

[37] Pulat G.O., Gokmen O., Cevik Z.B.Y., Karaman O. (2021). Role of functionalized self-assembled peptide hydrogels in in vitro vasculogenesis. Soft Matter.

[38] Ding C.-F., Lin Y.-C., Yang C.-C., Chang C.-M., Huang K.-C., Tseng S.-F., Lin K.-M., Hsiao W.-T. (2021). Surface Modification of Addition Manufactured Ti-6Al-4V Alloys by Ultraviolet Pulsed Laser Scanning Technique: Morphologies, Roughness And Electrical Properties. J. Laser Micro Nanoeng..

[39] Ahmadiyan S., Khalil-Allafi J., Etminanfar M.R., Safavi M.S., Hosseini M. (2022). Antibacterial activity and biocompatibility of Ag-coated Ti implants: Importance of surface modification parameters. Trans. Inst. Met. Finish..

[40] Bu Y., Ma J., Bei J., Wang S. (2019). Surface Modification of Aliphatic Polyester to Enhance Biocompatibility. Front. Bioeng. Biotechnol..

[41] Campisi M., Shin Y., Osaki T., Hajal C., Chiono V., Kamm R.D. (2018). 3D self-organized microvascular model of the human blood-brain barrier with endothelial cells, pericytes and astrocytes. Biomaterials.

[42] Zhang Z., Tang J., Song J., Xie M., Liu Y., Dong Z., Liu X., Li X., Zhang M., Chen Y. (2022). Elabela alleviates ferroptosis, myocardial remodeling, fibrosis and heart dysfunction in hypertensive mice by modulating the IL-6/STAT3/ GPX4 signaling. Free. Radic. Biol. Med..

[43] Tan W., Boodagh P., Selvakumar P.P., Keyser S. (2023). Strategies to counteract adverse remodeling of vascular graft: A 3D view of current graft innovations. Front. Bioeng. Biotechnol..

[44] van Haaften E.E., Wissing T.B., Kurniawan N.A., Smits A.I.P.M., Bouten C.V.C. (2020). Human In Vitro Model Mimicking Material-Driven Vascular Regeneration Reveals How Cyclic Stretch and Shear Stress Differentially Modulate Inflammation and Matrix Deposition. Adv. Biosyst..

[45] Yang L., Li X., Wu Y., Du P., Sun L., Yu Z., Song S., Yin J., Ma X., Jing C. (2020). Preparation of PU/Fibrin Vascular Scaffold with Good Biomechanical Properties and Evaluation of Its Performance in vitro and in vivo. Int. J. Nanomed..

[46] Yang Y., Lei D., Huang S., Yang Q., Song B., Guo Y., Shen A., Yuan Z., Li S., Qing F.-L. (2019). Elastic 3D-Printed Hybrid Polymeric Scaffold Improves Cardiac Remodeling after Myocardial Infarction. Adv. Healthc. Mater..

[47] Caracciolo P.C., Isabel Rial-Hermida M., Montini-Ballarin F., Abraham G.A., Concheiro A., Alvarez-Lorenzo C. (2017). Surface-modified bioresorbable electrospun scaffolds for improving hemocompatibility of vascular grafts. Mater. Sci. Eng. C-Mater. Biol. Appl..

[48] Davoudi P., Assadpour S., Derakhshan M.A., Ai J., Solouk A., Ghanbari H. (2017). Biomimetic modification of polyurethane-based nanofibrous vascular grafts: A promising approach towards stable endothelial lining. Mater. Sci. Eng. C-Mater. Biol. Appl..

[49] Mokhtari N., Kharazi A.Z. (2021). Blood compatibility and cell response improvement of poly glycerol sebacate/poly lactic acid scaffold for vascular graft applications. J. Biomed. Mater. Res. Part A.

[50] Fang J., Zhang J., Du J., Pan Y., Shi J., Peng Y., Chen W., Yuan L., Ye S.-H., Wagner W.R. (2016). Orthogonally Functionalizable Polyurethane with Subsequent Modification with Heparin and Endothelium-Inducing Peptide Aiming for Vascular Reconstruction. Acs Appl. Mater. Interfaces.

[51] Hashi C.K., Zhu Y., Yang G.-Y., Young W.L., Hsiao B.S., Wang K., Chu B., Li S. (2007). Antithrombogenic property of bone marrow mesenchymal stem cells in nanofibrous vascular grafts. Proc. Natl. Acad. Sci. USA.

[52] Lorentz K.L., Gupta P., Shehabeldin M.S., Cunnane E.M., Ramaswamy A.K., Verdelis K., Dileo M.V., Little S.R., Weinbaum J.S., Sfeir C.S. (2021). CCL2 loaded microparticles promote acute patency in silk-based vascular grafts implanted in rat aortae. Acta Biomater..

[53] Ariga K., Ahn E., Park M., Kim B.S. (2019). Layer-by-Layer Assembly: Recent Progress from Layered Assemblies to Layered Nanoarchitectonics. Chem. Asian J..

[54] Manivasagam V.K., Sabino R.M., Kantam P., Popat K.C. (2021). Surface modification strategies to improve titanium hemocompatibility: A comprehensive review. Mater. Adv..

[55] Jana S. (2019). Endothelialization of cardiovascular devices. Acta Biomater..

[56] Ren X., Feng Y., Guo J., Wang H., Li Q., Yang J., Hao X., Lv J., Ma N., Li W. (2015). Surface modification and endothelialization of biomaterials as potential scaffolds for vascular tissue engineering applications. Chem. Soc. Rev..

[57] Bai L., Zhao J., Wang M., Feng Y., Ding J. (2020). Matrix-Metalloproteinase-Responsive Gene Delivery Surface for Enhanced in Situ Endothelialization. Acs Appl. Mater. Interfaces.

[58] Zhao J., Feng Y. (2020). Surface Engineering of Cardiovascular Devices for Improved Hemocompatibility and Rapid Endothelialization. Adv. Healthc. Mater..

[59] Wang B., Hua J., You R., Yan K., Ma L. (2021). Electrochemically deposition of catechol-chitosan hydrogel coating on coronary stent with robust copper ions immobilization capability and improved interfacial biological activity. Int. J. Biol. Macromol..

[60] Yan K., Yang C., Zhong W., Lu Z., Li X., Shi X., Wang D. (2020). Wire templated electrodeposition of vessel-like structured chitosan hydrogel by using a pulsed electrical signal. Soft Matter.

[61] Meng S., Liu Z., Shen L., Guo Z., Chou L.L., Zhong W., Du Q., Ge J. (2009). The effect of a layer-by-layer chitosan-heparin coating on the endothelialization and coagulation properties of a coronary stent system. Biomaterials.

[62] Frank L.A., Onzi G.R., Morawski A.S., Pohlmann A.R., Guterres S.S., Contri R.V. (2020). Chitosan as a coating material for nanoparticles intended for biomedical applications. React. Funct. Polym..

[63] Wang H., Feng Y., Zhao H., Xiao R., Lu J., Zhang L., Guo J. (2012). Electrospun hemocompatible PU/gelatin-heparin nanofibrous bilayer scaffolds as potential artificial blood vessels. Macromol. Res..

[64] Wang H., Feng Y., Fang Z., Xiao R., Yuan W., Khan M. (2013). Fabrication and characterization of electrospun gelatin-heparin nanofibers as vascular tissue engineering. Macromol. Res..

[65] Yuan W., Feng Y., Wang H., Yang D., An B., Zhang W., Khan M., Guo J. (2013). Hemocompatible surface of electrospun nanofibrous scaffolds by ATRP modification. Mater. Sci. Eng. C-Mater. Biol. Appl..

[66] Maji K., Pramanik K. (2022). Electrospun scaffold for bone regeneration. Int. J. Polym. Mater. Polym. Biomater..

[67] Jiang C., Wang K., Liu Y., Zhang C., Wang B. (2021). Textile-based sandwich scaffold using wet electrospun yarns for skin tissue engineering. J. Mech. Behav. Biomed. Mater..

[68] Zhou W., Feng Y., Yang J., Fan J., Lv J., Zhang L., Guo J., Ren X., Zhang W. (2015). Electrospun scaffolds of silk fibroin and poly(lactide-co-glycolide) for endothelial cell growth. J. Mater. Sci.-Mater. Med..

[69] Shi C., Yuan W., Khan M., Li Q., Feng Y., Yao F., Zhang W. (2015). Hydrophilic PCU scaffolds prepared by grafting PEGMA and immobilizing gelatin to enhance cell adhesion and proliferation. Mater. Sci. Eng. C-Mater. Biol. Appl..

[70] Guo F., Guo Z., Gao L., Zheng L. (2021). Preparation and properties of thermal bonded fibrous artificial blood vessels. J. Text. Res..

[71] Hu J., Jian Z., Lu C., Liu N., Yue T., Lan W., Liu Y. (2021). New Method for Preparing Small-Caliber Artificial Blood Vessel with Controllable Microstructure on the Inner Wall Based on Additive Material Composite Molding. Micromachines.

[72] Yu L., Feng Y., Li Q., Hao X., Liu W., Zhou W., Shi C., Ren X., Zhang W. (2015). PLGA/SF blend scaffolds modified with plasmid complexes for enhancing proliferation of endothelial cells. React. Funct. Polym..

[73] Feng Y., Lu W., Ren X., Liu W., Guo M., Ullah I., Zhang W. (2016). Electrospun Poly(lactide-co-glycolide-co-3(S)-methyl-morpholine-2,5-dione) Nanofibrous Scaffolds for Tissue Engineering. Polymers.

[74] Feng Y., Liu W., Ren X., Lu W., Guo M., Behl M., Lendlein A., Zhang W. (2016). Evaluation of Electrospun PCL-PIBMD Meshes Modified with Plasmid Complexes in Vitro and in Vivo. Polymers.

[75] Bai L., Li Q., Duo X., Hao X., Zhang W., Shi C., Guo J., Ren X., Feng Y. (2017). Electrospun PCL-PIBMD/SF blend scaffolds with plasmid complexes for endothelial cell proliferation. RSC Adv..

[76] Chen Z., Liu Y., Huang J., Hao M., Hu X., Qian X., Fan J., Yang H., Yang B. (2022). Influences of Process Parameters of Near-Field Direct-Writing Melt Electrospinning on Performances of Polycaprolactone/Nano-Hydroxyapatite Scaffolds. Polymers.

[77] Zhao J., Bai L., Ren X.-k., Guo J., Xia S., Zhang W., Feng Y. (2019). Co-immobilization of ACH(11) antithrombotic peptide and CAG cell-adhesive peptide onto vascular grafts for improved hemocompatibility and endothelialization. Acta Biomater..

[78] Zhou J., Wang M., Wei T., Bai L., Zhao J., Wang K., Feng Y. (2021). Endothelial Cell-Mediated Gene Delivery for In Situ Accelerated Endothelialization of a Vascular Graft. Acs Appl. Mater. Interfaces.

[79] Bai L., Zhao J., Li Q., Guo J., Ren X., Xia S., Zhang W., Feng Y. (2019). Biofunctionalized Electrospun PCL-PIBMD/SF Vascular Grafts with PEG and Cell-Adhesive Peptides for Endothelialization. Macromol. Biosci..

[80] Chen Q., Wu J., Liu Y., Li Y., Zhang C., Qi W., Yeung K.W.K., Wong T.M., Zhao X., Pan H. (2019). Electrospun chitosan/PVA/bioglass Nanofibrous membrane with spatially designed structure for accelerating chronic wound healing. Mater. Sci. Eng. C-Mater. Biol. Appl..

[81] Wang M., Gu J., Hao Y., Qin X., Yu Y., Zhang H. (2022). Adhesive, sustained-release, antibacterial, cytocompatible hydrogel-based nanofiber membrane assembled from polysaccharide hydrogels and functionalized nanofibers. Cellulose.

[82] Miller R.J., Chan C.Y., Rastogi A., Grant A.M., White C.M., Bette N., Schaub N.J., Corey J.M. (2018). Combining electrospun nanofibers with cell-encapsulating hydrogel fibers for neural tissue engineering. J. Biomater. Sci.-Polym. Ed..

[83] Ulag S., Kalkandelen C., Oktar F.N., Uzun M., Sahin Y.M., Karademir B., Arslan S., Ozbolat I.T., Mahirogullari M., Gunduz O. (2019). 3D Printing Artificial Blood Vessel Constructs Using PCL/Chitosan/Hydrogel Biocomposites. Chemistryselect.

[84] Hann S.Y., Cui H., Chen G., Boehm M., Esworthy T., Zhang L.G. (2022). 3D printed biomimetic flexible blood vessels with iPS cell-laden hierarchical multilayers. Biomed. Eng. Adv..

[85] Wu X., Liu S., Chen K., Wang F., Feng C., Xu L., Zhang D. (2021). 3D printed chitosan-gelatine hydrogel coating on titanium alloy surface as biological fixation interface of artificial joint prosthesis. Int. J. Biol. Macromol..

[86] Peng X., Xu X., Deng Y., Xie X., Xu L., Xu X., Yuan W., Yang B., Yang X., Xia X. (2021). Ultrafast Self-Gelling and Wet Adhesive Powder for Acute Hemostasis and Wound Healing. Adv. Funct. Mater..

[87] Zhu Y., Kong L., Farhadi F., Xia W., Chang J., He Y., Li H. (2019). An injectable continuous stratified structurally and functionally biomimetic construct for enhancing osteochondral regeneration. Biomaterials.

[88] Malektaj H., Imani R., Siadati M.H. (2021). Study of injectable PNIPAAm hydrogels containing niosomal angiogenetic drug delivery system for potential cardiac tissue regeneration. Biomed. Mater..

[89] Zhang Y., Chen H., Zhang T., Zan Y., Ni T., Cao Y., Wang J., Liu M., Pei R. (2019). Injectable hydrogels from enzyme-catalyzed crosslinking as BMSCs-laden scaffold for bone repair and regeneration. Mater. Sci. Eng. C-Mater. Biol. Appl..

[90] Ge J., Li Y., Wang M., Gao C., Yang S., Lei B. (2021). Engineering conductive antioxidative antibacterial nanocomposite hydrogel scaffolds with oriented channels promotes structure-functional skeletal muscle regeneration. Chem. Eng. J..

[91] Im S.B., Tripathi G., Thi Thao Thanh L., Lee B.T. (2021). Early-stage bone regeneration of hyaluronic acid supplemented with porous 45s5 bioglass-derived granules: An injectable system. Biomed. Mater..

[92] Wang L., Li T., Wang Z., Hou J., Liu S., Yang Q., Yu L., Guo W., Wang Y., Guo B. (2022). Injectable remote magnetic nanofiber/hydrogel multiscale scaffold for functional anisotropic skeletal muscle regeneration. Biomaterials.

[93] Sarviya N., Basu S.M., Induvahi V., Giri J. (2022). Laponite-Gelatin Nanofibrous Microsphere Promoting Human Dental Follicle Stem Cells Attachment and Osteogenic Differentiation for Noninvasive Stem Cell Transplantation. Macromol. Biosci..

[94] Abd El-Fattah A., Mansour A. (2018). Viscoelasticity, mechanical properties, and in vitro biodegradation of injectable chitosan-poly(3-hydroxybutyrate-co-3-hydroxyvalerate)/nanohydroxyapatite composite hydrogel. Bull. Mater. Sci..

[95] Lee S.J., Nah H., Heo D.N., Kim K.-H., Seok J.M., Heo M., Moon H.-J., Lee D., Lee J.S., An S.Y. (2020). Induction of osteogenic differentiation in a rat calvarial bone defect model using an In situ forming graphene oxide incorporated glycol chitosan/oxidized hyaluronic acid injectable hydrogel. Carbon.

[96] Kazemi-Aghdam F., Jahed V., Dehghan-Niri M., Ganji F., Vasheghani-Farahani E. (2021). Injectable chitosan hydrogel embedding modified halloysite nanotubes for bone tissue engineering. Carbohydr. Polym..

[97] Baysan G., Gunes O.C., Akokay P., Husemoglu R.B., Ertugruloglu P., Albayrak A.Z., Cecen B., Havitcioglu H. (2022). Loofah-chitosan and poly (-3-hydroxybutyrate-co-3-hydroxyvalerate) (PHBV) based hydrogel scaffolds for meniscus tissue engineering applications. Int. J. Biol. Macromol..

[98] Kuang W., Liu C., Xu H. (2021). The application of decellularized nucleus pulposus matrix/chitosan with transforming growth factor beta 3 for nucleus pulposus tissue engineering. Cytotechnology.

[99] Zhou Y., Zhao S., Zhang C., Liang K., Li J., Yang H., Gu S., Bai Z., Ye D., Xu W. (2018). Photopolymerized maleilated chitosan/thiol-terminated poly (vinyl alcohol) hydrogels as potential tissue engineering scaffolds. Carbohydr. Polym..

[100] Castro A.P.G., Ruben R.B., Goncalves S.B., Pinheiro J., Guedes J.M., Fernandes P.R. (2019). Numerical and experimental evaluation of TPMS Gyroid scaffolds for bone tissue engineering. Comput. Methods Biomech. Biomed. Eng..

[101] Rouhollahi A., Ilegbusi O., Foroosh H. (2021). Segmentation and Pore Structure Estimation in SEM Images of Tissue Engineering Scaffolds Using Genetic Algorithm. Ann. Biomed. Eng..

[102] Puppi D., Chiellini F. (2021). Computer-Aided Wet-Spinning. Methods Mol. Biol..

[103] Calore A.R., Sinha R., Harings J., Bernaerts K.V., Mota C., Moroni L. (2021). Additive Manufacturing Using Melt Extruded Thermoplastics for Tissue Engineering. Methods Mol. Biol..

[104] Forget A., Rojas D., Waibel M., Pencko D., Gunenthiran S., Ninan N., Loudovaris T., Drogemuller C., Coates P.T., Voelcker N.H. (2020). Facile preparation of tissue engineering scaffolds with pore size gradients using the muesli effect and their application to cell spheroid encapsulation. J. Biomed. Mater. Res. Part B-Appl. Biomater..

[105] Li G., Qin S., Liu X., Zhang D., He M. (2019). Structure and properties of nano-hydroxyapatite/poly(butylene succinate) porous scaffold for bone tissue engineering prepared by using ethanol as porogen. J. Biomater. Appl..

[106] Chen W., Jiang K., Mack A., Sachok B., Zhu X., Barber W.E., Wang X. (2015). Synthesis and optimization of wide pore superficially porous particles by a one-step coating process for separation of proteins and monoclonal antibodies. J. Chromatogr. A.

[107] Allijn I., du Preez N., Tasior M., Bansal R., Stamatialis D. (2022). One-Step Fabrication of Porous Membrane-Based Scaffolds by Air-Water Interfacial Phase Separation: Opportunities for Engineered Tissues. Membranes.

[108] Lopresti F., Liga A., Capuana E., Gulfi D., Zanca C., Inguanta R., Brucato V., La Carrubba V., Pavia F.C. (2022). Effect of Polyhydroxyalkanoate (PHA) Concentration on Polymeric Scaffolds Based on Blends of Poly-L-Lactic Acid (PLLA) and PHA Prepared via Thermally Induced Phase Separation (TIPS). Polymers.

[109] Salehi M., Farzamfar S., Bozorgzadeh S., Bastami F. (2019). Fabrication of Poly(L-Lactic Acid)/Chitosan Scaffolds by Solid-Liquid Phase Separation Method for Nerve Tissue Engineering: An In Vitro Study on Human Neuroblasts. J. Craniofacial Surg..

[110] Brougham C.M., Levingstone T.J., Shen N., Cooney G.M., Jockenhoevel S., Flanagan T.C., O’Brien F.J. (2017). Freeze-Drying as a Novel Biofabrication Method for Achieving a Controlled Microarchitecture within Large, Complex Natural Biomaterial Scaffolds. Adv. Healthc. Mater..

[111] Manavitehrani I., Le T.Y.L., Daly S., Wang Y., Maitz P.K., Schindeler A., Dehghani F. (2019). Formation of porous biodegradable scaffolds based on poly(propylene carbonate) using gas foaming technology. Mater. Sci. Eng. C-Mater. Biol. Appl..

[112] Chen Y., Jia Z., Shafiq M., Xie X., Xiao X., Castro R., Rodrigues J., Wu J., Zhou G., Mo X. (2021). Gas foaming of electrospun poly(L-lactide-co-caprolactone)/silk fibroin nanofiber scaffolds to promote cellular infiltration and tissue regeneration. Colloids Surf. B-Biointerfaces.

[113] Lv Q., Ma C. (2022). A novel protocol for injectable artificial cartilage constructs based on programmed shape-morphing hydrogels for cartilage regeneration. Chem. Eng. J..

[114] Valente S.A., Silva L.M., Lopes G.R., Sarmento B., Coimbra M.A., Passos C.P. (2022). Polysaccharide-based formulations as potential carriers for pulmonary delivery—A review of their properties and fates. Carbohydr. Polym..

[115] Alves N.M., Mano J.F. (2008). Chitosan derivatives obtained by chemical modifications for biomedical and environmental applications. Int. J. Biol. Macromol..

[116] Leite A.J., Costa R.R., Costa A.M.S., Maciel J.S., Costa J.F.G., de Paula R.C.M., Mano J.F. (2017). The potential of cashew gum functionalization as building blocks for layer-by-layer films. Carbohydr. Polym..

[117] Chung Y.C., Wang H.L., Chen Y.M., Li S.L. (2003). Effect of abiotic factors on the antibacterial activity of chitosan against waterborne pathogens. Bioresour. Technol..

[118] Bingjun Q., Jung J., Zhao Y. (2015). Impact of Acidity and Metal Ion on the Antibacterial Activity and Mechanisms of beta- and alpha-Chitosan. Appl. Biochem. Biotechnol..

[119] Li J., Tian X., Hua T., Fu J., Koo M., Chan W., Poon T. (2021). Chitosan Natural Polymer Material for Improving Antibacterial Properties of Textiles. Acs Appl. Bio Mater..

[120] Ali A., Ahmed S. (2018). A review on chitosan and its nanocomposites in drug delivery. Int. J. Biol. Macromol..

[121] Elgadir M.A., Uddin M.S., Ferdosh S., Adam A., Chowdhury A.J.K., Sarker M.Z.I. (2015). Impact of chitosan composites and chitosan nanoparticle composites on various drug delivery systems: A review. J. Food Drug Anal..

[122] Pinheiro A.C., Bourbon A.I., Cerqueira M.A., Maricato E., Nunes C., Coimbra M.A., Vicente A.A. (2015). Chitosan/fucoidan multilayer nanocapsules as a vehicle for controlled release of bioactive compounds. Carbohydr. Polym..

[123] Pistone A., Iannazzo D., Celesti C., Scolaro C., Giofre S.V., Romeo R., Visco A. (2020). Chitosan/PAMAM/Hydroxyapatite Engineered Drug Release Hydrogels with Tunable Rheological Properties. Polymers.

[124] Wang Y., Liu S., Yu W. (2020). Bioinspired Anisotropic Chitosan Hybrid Hydrogel. Acs Appl. Bio Mater..

[125] Jiao Y., Niu L.N., Ma S., Li J., Tay F.R., Chen J.H. (2017). Quaternary ammonium-based biomedical materials: State-of-the-art, toxicological aspects and antimicrobial resistance. Prog. Polym. Sci..

[126] Wu Y., Wang H., Fan C., Xu Z., Liu B., Liu W. (2020). A smart indwelling needle with on-demand switchable anticoagulant and hemostatic activities. Mater. Horiz..

[127] do Nascimento E.G., de Caland L.B., de Medeiros A.S.A., Fernandes-Pedrosa M.F., Soares-Sobrinho J.L., dos Santos K.S.C.R., da Silva-Junior A.A. (2017). Tailoring Drug Release Properties by Gradual Changes in the Particle Engineering of Polysaccharide Chitosan Based Powders. Polymers.

[128] Kashyap P.L., Xiang X., Heiden P. (2015). Chitosan nanoparticle based delivery systems for sustainable agriculture. Int. J. Biol. Macromol..

[129] Raza Z.A., Khalil S., Ayub A., Banat I.M. (2020). Recent developments in chitosan encapsulation of various active ingredients for multifunctional applications. Carbohydr. Res..

[130] Avcu E., Bastan F.E., Abdullah H.Z., Rehman M.A.U., Avcu Y.Y., Boccaccini A.R. (2019). Electrophoretic deposition of chitosan-based composite coatings for biomedical applications: A review. Prog. Mater. Sci..

[131] Wei Z., Pan P., Hong F.F., Cao Z., Ji Y., Chen L. (2021). A novel approach for efficient fabrication of chitosan nanoparticles-embedded bacterial nanocellulose conduits. Carbohydr. Polym..

[132] Ciobanu B.C., Cadinoiu A.N., Popa M., Desbrieres J., Peptu C.A. (2014). Modulated release from liposomes entrapped in chitosan/gelatin hydrogels. Mater. Sci. Eng. C-Mater. Biol. Appl..

[133] Qi R.-Q., Liu W., Wang D.-Y., Meng F.-Q., Wang H.-Y., Qi H.-Y. (2021). Development of local anesthetic drug delivery system by administration of organo-silica nanoformulations under ultrasound stimuli: In vitro and in vivo investigations. Drug Deliv..

[134] Cao R., Yu H., Long H., Zhang H., Hao C., Shi L., Du Y., Jiao S., Guo A., Ma L. (2022). Low deacetylation degree chitosan oligosaccharide protects against IL-1 beta induced inflammation and enhances autophagy activity in human chondrocytes. J. Biomater. Sci.-Polym. Ed..

[135] Kang M.-L., Kim J.-E., Im G.-I. (2016). Thermoresponsive nanospheres with independent dual drug release profiles for the treatment of osteoarthritis. Acta Biomater..

[136] Li S., Liu J., Liu S., Jiao W., Wang X. (2021). Chitosan oligosaccharides packaged into rat adipose mesenchymal stem cells-derived extracellular vesicles facilitating cartilage injury repair and alleviating osteoarthritis. J. Nanobiotechnology.

[137] Wang W.-D., Chen C., Fu X. (2020). Glycation mechanism of lactoferrin-chitosan oligosaccharide conjugates with improved antioxidant activity revealed by high-resolution mass spectroscopy. Food Funct..

[138] Zhao D., Wang J., Tan L., Sun C., Dong J. (2013). Synthesis of N-furoyl chitosan and chito-oligosaccharides and evaluation of their antioxidant activity in vitro. Int. J. Biol. Macromol..

[139] Bilal M., Nunes L.V., Saviatto Duarte M.T., Romanholo Ferreira L.F., Soriano R.N., Iqbal H.M.N. (2021). Exploitation of Marine-Derived Robust Biological Molecules to Manage Inflammatory Bowel Disease. Mar. Drugs.

[140] Rajendran S.R.C.K., Okolie C.L., Udenigwe C.C., Mason B. (2017). Structural features underlying prebiotic activity of conventional and potential prebiotic oligosaccharides in food and health. J. Food Biochem..

[141] Sutthasupha P., Promsan S., Thongnak L., Pengrattanachot N., Phengpol N., Jaruan O., Jaikumkao K., Muanprasat C., Pichyangkura R., Chatsudthipong V. (2022). Chitosan oligosaccharide mitigates kidney injury in prediabetic rats by improving intestinal barrier and renal autophagy. Carbohydr. Polym..

[142] Wang S., Ji X., Chen S., Zhang C., Wang Y., Lin H., Zhao L. (2022). Study of double-bonded carboxymethyl chitosan/cysteamine-modified chondroitin sulfate composite dressing for hemostatic application. Eur. Polym. J..

[143] Xia L., Wang S., Jiang Z., Chi J., Yu S., Li H., Zhang Y., Li L., Zhou C., Liu W. (2021). Hemostatic performance of chitosan-based hydrogel and its study on biodistribution and biodegradability in rats. Carbohydr. Polym..

[144] Zhang W., Xin Y., Yin B., Ye G.L., Wang J.X., Shen J.F., Li L., Yang Q.H. (2019). Synthesis and properties of crosslinked carboxymethyl chitosan and its hemostatic and wound healing effects on liver injury of rats. J. Biomater. Appl..

[145] Gao Y., Zhang X., Jin X. (2019). Preparation and Properties of Minocycline-Loaded Carboxymethyl Chitosan Gel/Alginate Nonwovens Composite Wound Dressings. Mar. Drugs.

[146] He Y., Li Y., Sun Y., Zhao S., Feng M., Xu G., Zhu H., Ji P., Mao H., He Y. (2021). A double-network polysaccharide-based composite hydrogel for skin wound healing. Carbohydr. Polym..

[147] Soubhagya A.S., Balagangadharan K., Selvamurugan N., Seeli D.S., Prabaharan M. (2022). Preparation and characterization of chitosan/carboxymethyl pullulan/bioglass composite films for wound healing. J. Biomater. Appl..

[148] Cheng H., Shi W., Feng L., Bao J., Chen Q., Zhao W., Zhao C. (2021). Facile and green approach towards biomass-derived hydrogel powders with hierarchical micro-nanostructures for ultrafast hemostasis. J. Mater. Chem. B.

[149] Chien Y., Liao Y.-W., Liu D.-M., Lin H.-L., Chen S.-J., Chen H.-L., Peng C.-H., Liang C.-M., Mou C.-Y., Chiou S.-H. (2012). Corneal repair by human corneal keratocyte-reprogrammed iPSCs and amphiphatic carboxymethyl-hexanoyl chitosan hydrogel. Biomaterials.

[150] Malik M.H., Shahzadi L., Batool R., Safi S.Z., Khan A.S., Khan A.F., Chaudhry A.A., Rehman I.U., Yar M. (2020). Thyroxine-loaded chitosan/carboxymethyl cellulose/hydroxyapatite hydrogels enhance angiogenesis in in-ovo experiments. Int. J. Biol. Macromol..

[151] Medeiros Borsagli F.G.L., de Souza A.J.M., Paiva A.E. (2020). Ecofriendly multifunctional thiolated carboxymethyl chitosan-based 3D scaffolds with luminescent properties for skin repair and theragnostic of tissue regeneration. Int. J. Biol. Macromol..

[152] Oliveira D.M.L., Rezende P.S., Barbosa T.C., Andrade L.N., Bani C., Tavares D.S., da Silva C.F., Chaud M.V., Padilha F., Cano A. (2020). Double membrane based on lidocaine-coated polymyxin-alginate nanoparticles for wound healing: In vitro characterization and in vivo tissue repair. Int. J. Pharm..

[153] Barros S.C., da Silva A.A., Costa D.B., Costa C.M., Lanceros-Mendez S., Tamano Maciavello M.N., Gomez Ribelles J.L., Sentanin F., Pawlicka A., Silva M.M. (2015). Thermal-mechanical behaviour of chitosan-cellulose derivative thermoreversible hydrogel films. Cellulose.

[154] Berardesca E., Iorizzo M., Abril E., Guglielmini G., Caserini M., Palmieri R., Pierard G.E. (2012). Clinical and instrumental assessment of the effects of a new product based on hydroxypropyl chitosan and potassium azeloyl diglycinate in the management of rosacea. J. Cosmet. Dermatol..

[155] Park J.-M., Kim M., Park H.-S., Jang A., Min J., Kim Y.-H. (2013). Immobilization of lysozyme-CLEA onto electrospun chitosan nanofiber for effective antibacterial applications. Int. J. Biol. Macromol..

[156] Ding F., You J., Weng X., Zhou J., Zhang X., Zhou X., Zhang L. (2012). Exploring Quaternized Hydroxyethylcellulose as Potential Gene Carriers. Chin. J. Chem..

[157] Faizuloev E., Marova A., Nikonova A., Volkova I., Gorshkova M., Izumrudov V. (2012). Water-soluble N- (2-hydroxy-3-trimethylammonium)propyl chitosan chloride as a nucleic acids vector for cell transfection. Carbohydr. Polym..

[158] Yu S., Hao S., Sun B., Zhao D., Yan X., Jin Z., Zhao K. (2020). Quaternized Chitosan Nanoparticles in Vaccine Applications. Curr. Med. Chem..

[159] Chen Q., Xiao S., Shi S.Q., Cai L. (2020). Synthesis, Characterization, and Antibacterial Activity of N-substituted Quaternized Chitosan and Its Cellulose-based Composite Film. Bioresources.

[160] Jang S.-C., Chuang F.-S., Tsen W.-C., Kuo T.-W. (2019). Quaternized chitosan/functionalized carbon nanotubes composite anion exchange membranes. J. Appl. Polym. Sci..

[161] Rahimi M., Ahmadi R., Kafil H.S., Shafiei-Irannejad V. (2019). A novel bioactive quaternized chitosan and its silver-containing nanocomposites as a potent antimicrobial wound dressing: Structural and biological properties. Mater. Sci. Eng. C-Mater. Biol. Appl..

[162] Drozd N.N., Logvinova Y.S., Shagdarova B.T., Il’ina A.V., Varlamov V.P. (2019). Analysis of the Action of Quaternized Chitosans with Different Molecular Weight on Anticoagulant Activity of Heparins In Vitro. Bull. Exp. Biol. Med..

[163] Han G., Xia X., Pan Z., Lin Y., Li L., Jiao Y., Zhou C., Ding S. (2020). Different influence of sulfated chitosan with different sulfonic acid group sites on HUVECs behaviors. J. Biomater. Sci.-Polym. Ed..

[164] Wang T., Zhou Y., Xie W., Chen L., Zheng H., Fan L. (2012). Preparation and anticoagulant activity of N-succinyl chitosan sulfates. Int. J. Biol. Macromol..

[165] Zhang S., Chen J., Yu Y., Dai K., Wang J., Liu C. (2019). Accelerated Bone Regenerative Efficiency by Regulating Sequential Release of BMP-2 and VEGF and Synergism with Sulfated Chitosan. Acs Biomater. Sci. Eng..

[166] Cevher E., Salomon S.K., Makrakis A., Li X.W., Brocchini S., Alpar H.O. (2015). Development of chitosan-pullulan composite nanoparticles for nasal delivery of vaccines: Optimisation and cellular studies. J. Microencapsul..

[167] Zaki N.M., Mortada N.D., Awad G.A.S., ElHady S.S.A. (2006). Rapid-onset intranasal delivery of metoclopramide hydrochloride—Part II: Safety of various absorption enhancers and pharmacokinetic evaluation. Int. J. Pharm..

[168] Zhang S., Huang S., Lu L., Song X., Li P., Wang F. (2018). Curdlan sulfate-O-linked quaternized chitosan nanoparticles: Potential adjuvants to improve the immunogenicity of exogenous antigens via intranasal vaccination. Int. J. Nanomed..

[169] Dong C., Chen W., Liu C. (2014). Flocculation of algal cells by amphoteric chitosan-based flocculant. Bioresour. Technol..

[170] Rasad M.S.B.A., Halim A.S., Hashim K., Rashid A.H.A., Yusof N., Shamsuddin S. (2010). In vitro evaluation of novel chitosan derivatives sheet and paste cytocompatibility on human dermal fibroblasts. Carbohydr. Polym..

[171] Zhu L., Fan Z.-Q., Shi X.-Q., Wang N., Bo Y.-Y., Guo H.-E. (2020). A novel silkworm pupae carboxymethyl chitosan inhibits mouse L929 fibroblast proliferation. Scienceasia.

[172] Wang J., Zhuang S. (2022). Chitosan-based materials: Preparation, modification and application. J. Clean. Prod..

[173] Jayakumar R., Prabaharan M., Nair S.V., Tokura S., Tamura H., Selvamurugan N. (2010). Novel carboxymethyl derivatives of chitin and chitosan materials and their biomedical applications. Prog. Mater. Sci..

[174] Shariatinia Z. (2018). Carboxymethyl chitosan: Properties and biomedical applications. Int. J. Biol. Macromol..

[175] Subhapradha N., Ramasamy P., Srinivasan A., Madeswaran P., Shanmugam V., Shanmugam A. (2013). Sulfation of beta-chitosan and evaluation of biological activity from gladius of Sepioteuthis lessoniana. Int. J. Biol. Macromol..

[176] Ngo D.-H., Kim S.-K. (2014). Antioxidant effects of chitin, chitosan, and their derivatives. Adv. Food Nutr. Res..

[177] Skorik Y.A., Kritchenkov A.S., Moskalenko Y.E., Golyshev A.A., Raik S.V., Whaley A.K., Vasina L.V., Sonin D.L. (2017). Synthesis of N-succinyl- and N-glutaryl-chitosan derivatives and their antioxidant, antiplatelet, and anticoagulant activity. Carbohydr. Polym..

[178] Xing R.E., Liu S., Guo Z.Y., Yu H.H., Wang P.B., Li C.P., Li Z., Li P.C. (2005). Relevance of molecular weight of chitosan and its derivatives and their antioxidant activities in vitro. Bioorganic Med. Chem..

[179] Aguanell A., del Pozo M.L., Perez-Martin C., Pontes G., Bastida A., Fernandez-Mayoralas A., Garcia-Junceda E., Revuelta J. (2022). Chitosan sulfate-lysozyme hybrid hydrogels as platforms with fine-tuned and sustained inherent antibiotic and antioxidant activities. Carbohydr. Polym..

[180] Luo P., Nie M., Wen H., Xu W., Fan L., Cao Q. (2018). Preparation and characterization of carboxymethyl chitosan sulfate/oxidized konjac glucomannan hydrogels. Int. J. Biol. Macromol..

[181] Ponsubha S., Jaiswal A.K. (2020). Effect of interpolymer complex formation between chondroitin sulfate and chitosan-gelatin hydrogel on physico-chemical and rheological properties. Carbohydr. Polym..

[182] Afgan S., Yadav P., Jaiswal S., Pal K., Kumar R., Singh V., Koch B. (2022). Development of chitosan towards the self-healing and mechanically stronger biocompatible hydrogel. J. Indian Chem. Soc..

[183] Wang Y., Garcia C.R., Ding Z., Gabrilska R., Rumbaugh K.P., Wu J., Liu Q., Li W. (2020). Adhesive, Self-Healing, and Antibacterial Chitosan Hydrogels with Tunable Two-Layer Structures. ACS Sustain. Chem. Eng..

[184] Wang J., Li K., Xu J., Liu M., Li P., Li X., Fan Y. (2021). A biomimetic hierarchical small intestinal submucosa-chitosan sponge/chitosan hydrogel scaffold with a micro/nano structure for dural repair. J. Mater. Chem. B.

[185] Zhou R., Zhou Y., Cheng J., Cao J., Li M., Yu H., Wei D., Li B., Wang Y., Zhou Y. (2022). Surface configuration of microarc oxidized Ti with regionally loaded chitosan hydrogel containing ciprofloxacin for improving biological performance. Mater. Today Bio.

[186] Rahmi, Lelifajri, Nurfatimah R. (2018). Preparation of polyethylene glycol diglycidyl ether (PEDGE) crosslinked chitosan/activated carbon composite film for Cd^2+^ removal. Carbohydr. Polym..

[187] Bi S., Pang J., Huang L., Sun M., Cheng X., Chen X. (2020). The toughness chitosan-PVA double network hydrogel based on alkali solution system and hydrogen bonding for tissue engineering applications. Int. J. Biol. Macromol..

[188] Kim U.-J., Kim H.J., Choi J.W., Kimura S., Wada M. (2017). Cellulose-chitosan beads crosslinked by dialdehyde cellulose. Cellulose.

[189] Song X., Wang K., Tang C.-Q., Yang W.-W., Zhao W.-F., Zhao C.-S. (2018). Design of Carrageenan-Based Heparin-Mimetic Gel Beads as Self-Anticoagulant Hemoperfusion Adsorbents. Biomacromolecules.

[190] Hu L., Sun Y., Wu Y. (2013). Advances in chitosan-based drug delivery vehicles. Nanoscale.

[191] Ciric A., Medarevic D., Calija B., Dobricic V., Rmandic M., Barudzija T., Malenovic A., Djekic L. (2021). Effect of ibuprofen entrapment procedure on physicochemical and controlled drug release performances of chitosan/xanthan gum polyelectrolyte complexes. Int. J. Biol. Macromol..

[192] Sun H., Wang Y., Wang Y., Ji F., Wang A., Yang M., He X., Li L. (2022). Bivalent Regulation and Related Mechanisms of H3K4/27/9me3 in Stem Cells. Stem Cell Rev. Rep..

[193] Zhang Y.-x., Yang J.-w., Wu Y.-y., Hu X.-q., Zhang H.-b. (2021). The stability improvement of dextransucrase by artificial extension modification of the V domain of the enzyme. Enzym. Microb. Technol..

[194] Li Q., Cui J., Huang H., Yue Z., Chang Y., Li N., Han Z., Han Z.-c., Guo Z., Li Z. (2020). IGF-1C domain-modified chitosan hydrogel accelerates cutaneous wound healing by promoting angiogenesis. Future Med. Chem..

[195] Dai L., Cheng T., Duan C., Zhao W., Zhang W., Zou X., Aspler J., Ni Y. (2019). 3D printing using plant-derived cellulose and its derivatives: A review. Carbohydr. Polym..

[196] Rajabi M., McConnell M., Cabral J., Ali M.A. (2021). Chitosan hydrogels in 3D printing for biomedical applications. Carbohydr. Polym..

[197] Zhou L., Ramezani H., Sun M., Xie M., Nie J., Lv S., Cai J., Fu J., He Y. (2020). 3D printing of high-strength chitosan hydrogel scaffolds without any organic solvents. Biomater. Sci..

[198] Liu H., Yang X., Cheng X., Zhao G., Zheng G., Li X., Dong R. (2021). Theoretical and Experimental Research on Multi-Layer Vessel-like Structure Printing Based on 3D Bio-Printing Technology. Micromachines.

[199] Feng Y., Gao H.-L., Wu D., Weng Y.-T., Wang Z.-Y., Yu S.-H., Wang Z. (2021). Biomimetic Lamellar Chitosan Scaffold for Soft Gingival Tissue Regeneration. Adv. Funct. Mater..

[200] Tong J., Yang C., Qi L., Zhang J., Deng H., Du Y., Shi X. (2022). Tubular chitosan hydrogels with a tuneable lamellar structure programmed by electrical signals. Chem. Commun..

[201] Maleki S., Shamloo A., Kalantarnia F. (2022). Tubular TPU/SF nanofibers covered with chitosan-based hydrogels as small-diameter vascular grafts with enhanced mechanical properties. Sci. Rep..

[202] Liu Q., Ji N., Xiong L., Sun Q. (2020). Rapid gelling, self-healing, and fluorescence-responsive chitosan hydrogels formed by dynamic covalent crosslinking. Carbohydr. Polym..

[203] Pettinelli N., Rodriguez-Llamazares S., Abella V., Barral L., Bouza R., Farrag Y., Lago F. (2019). Entrapment of chitosan, pectin or kappa-carrageenan within methacrylate based hydrogels: Effect on swelling and mechanical properties. Mater. Sci. Eng. C-Mater. Biol. Appl..

[204] Yang Y., Wang X., Wu D. (2021). Chitosan-Based High-Mechanical Double-Network Hydrogels: Construction, Modulation and Applications. Acta Chim. Sin..

[205] Peng W., Li D., Dai K., Wang Y., Song P., Li H., Tang P., Zhang Z., Li Z., Zhou Y. (2022). Recent progress of collagen, chitosan, alginate and other hydrogels in skin repair and wound dressing applications. Int. J. Biol. Macromol..

[206] Ma Y., Zhou L., Yang C., Wang L., Yi S., Tong X., Xiao B., Chen J. (2021). Comparison of Sericins from Different Sources as Natural Therapeutics against Ulcerative Colitis. Acs Biomater. Sci. Eng..

[207] Adali T., Kalkan R., Karimizarandi L. (2019). The chondrocyte cell proliferation of a chitosan/silk fibroin/egg shell membrane hydrogels. Int. J. Biol. Macromol..

[208] Herron C., Hastings C.L., Herron-Rice C., Kelly H.M., O’Dwyer J., Duffy G.P. (2021). A Thermoresponsive Chitosan/beta-Glycerophosphate Hydrogel for Minimally Invasive Treatment of Critical Limb Ischaemia. Polymers.

[209] Hsieh F.-Y., Tao L., Wei Y., Hsu S.-h. (2017). A novel biodegradable self-healing hydrogel to induce blood capillary formation. Npg Asia Mater..

[210] Cui L., Li J., Guan S., Zhang K., Zhang K., Li J. (2022). Injectable multifunctional CMC/HA-DA hydrogel for repairing skin injury. Mater. Today Bio.

[211] Li Y., Li J., Shi Z., Wang Y., Song X., Wang L., Han M., Du H., He C., Zhao W. (2020). Anticoagulant chitosan-kappa-carrageenan composite hydrogel sorbent for simultaneous endotoxin and bacteria cleansing in septic blood. Carbohydr. Polym..

[212] Wang L., Gong T., Brown Z., Gu Y., Teng K., Ye W., Ming W. (2020). Preparation of Ascidian-Inspired Hydrogel Thin Films to Selectively Induce Vascular Endothelial Cell and Smooth Muscle Cell Growth. Acs Appl. Bio Mater..

[213] Vedadghavami A., Minooei F., Mohammadi M.H., Khetani S., Kolahchi A.R., Mashayekhan S., Sanati-Nezhad A. (2017). Manufacturing of hydrogel biomaterials with controlled mechanical properties for tissue engineering applications. Acta Biomater..

[214] Deng P., Liang X., Chen F., Chen Y., Zhou J. (2022). Novel multifunctional dual-dynamic-bonds crosslinked hydrogels for multi-strategy therapy of MRSA-infected wounds. Appl. Mater. Today.

[215] Yu F., Khan A.u.R., Li Y., Zhao B., Xie X., El-Newehy M., El-Hamshary H., Morsi Y., Li J., Pan J. (2022). A multifunctional nanofiber reinforced photo-crosslinking hydrogel for skin wound healing. Compos. Part B-Eng..

[216] Zhao F., Liu Y., Song T., Zhang B., Li D., Xiao Y., Zhang X. (2022). A chitosan-based multifunctional hydrogel containing in situ rapidly bioreduced silver nanoparticles for accelerating infected wound healing. J. Mater. Chem. B.

[217] Qu J., Li J., Zhu W., Xu Y., Zhou S., Yang Y., Qian X. (2022). Hybrid nanocomposite multinetwork hydrogel containing magnesium hydroxide nanoparticles with enhanced antibacterial activity for wound dressing applications. Polymer.

[218] Fonseca J.d.M., Medeiros S.d.F., Alves G.M., dos Santos D.M., Campana-Filho S.P., dos Santos A.M. (2019). Chitosan microparticles embedded with multi-responsive poly(N-vinylcaprolactam-co-itaconic acid-co-ethylene-glycol dimethacrylate)-based hydrogel nanoparticles as a new carrier for delivery of hydrophobic drugs. Colloids Surf. B-Biointerfaces.

[219] Yang C., Huang H., Fan S., Yang C., Chen Y., Yu B., Li W., Liao J. (2021). A Novel Dual-Crosslinked Functional Hydrogel Activated by POSS for Accelerating Wound Healing. Adv. Mater. Technol..

[220] Huang L., Zhu Z., Wu D., Gan W., Zhu S., Li W., Tian J., Li L., Zhou C., Lu L. (2019). Antibacterial poly (ethylene glycol) diacrylate/chitosan hydrogels enhance mechanical adhesiveness and promote skin regeneration. Carbohydr. Polym..

[221] Ara C., Jabeen S., Afshan G., Farooq A., Akram M.S., Asmatullah, Islam A., Ziafat S., Nawaz B., Khan R.U. (2022). Angiogenic potential and wound healing efficacy of chitosan derived hydrogels at varied concentrations of APTES in chick and mouse models. Int. J. Biol. Macromol..

[222] Li C., Jiang T., Zhou C., Jiang A., Lu C., Yang G., Nie J., Wang F., Yang X., Chen Z. (2023). Injectable self-healing chitosan-based POSS-PEG hybrid hydrogel as wound dressing to promote diabetic wound healing. Carbohydr. Polym..

[223] Bao C., Zhang X., Shen J., Li C., Zhang J., Feng X. (2022). Freezing-triggered gelation of quaternized chitosan reinforced with microfibrillated cellulose for highly efficient removal of bilirubin. J. Mater. Chem. B.

[224] Hao Y., Zhao W., Zhang L., Zeng X., Sun Z., Zhang D., Shen P., Li Z., Han Y., Li P. (2020). Bio-multifunctional alginate/chitosan/fucoidan sponges with enhanced angiogenesis and hair follicle regeneration for promoting full-thickness wound healing. Mater. Des..

[225] Wang X., Su B., Gao B., Zhou J., Ren X.-k., Guo J., Xia S., Zhang W., Feng Y. (2020). Cascaded bio-responsive delivery of eNOS gene and ZNF(580) gene to collaboratively treat hindlimb ischemia via pro-angiogenesis and anti-inflammation. Biomater. Sci..

[226] Zhang Q., Gao B., Muhammad K., Zhang X., Ren X.-k., Guo J., Xia S., Zhang W., Feng Y. (2019). Multifunctional gene delivery systems with targeting ligand CAGW and charge reversal function for enhanced angiogenesis. J. Mater. Chem. B.

[227] Li Q., Hao X., Zaidi S.S.A., Guo J., Ren X., Shi C., Zhang W., Feng Y. (2018). Oligohistidine and targeting peptide functionalized TAT-NLS for enhancing cellular uptake and promoting angiogenesis in vivo. J. Nanobiotechnology.

[228] Gao B., Zhang Q., Muhammad K., Ren X., Guo J., Xia S., Zhang W., Feng Y. (2019). A progressively targeted gene delivery system with a pH triggered surface charge-switching ability to drive angiogenesis in vivo. Biomater. Sci..

[229] Wang J., Zaidi S.S.A., Hasnain A., Guo J., Ren X., Xia S., Zhang W., Feng Y. (2018). Multitargeting Peptide-Functionalized Star-Shaped Copolymers with Comblike Structure and a POSS-Core To Effectively Transfect Endothelial Cells. Acs Biomater. Sci. Eng..

[230] Yang J., Hao X., Li Q., Akpanyung M., Nejjari A., Neve A.L., Ren X., Guo J., Feng Y., Shi C. (2017). CAGW Peptide- and PEG-Modified Gene Carrier for Selective Gene Delivery and Promotion of Angiogenesis in HUVECs in Vivo. Acs Appl. Mater. Interfaces.

[231] Gao B., Wang X., Wang M., Ren X.-k., Guo J., Xia S., Zhang W., Feng Y. (2021). “Green process” inspires gene delivery: Establishing positive feedback between CO_2_-enhanced bioactive carrier and gene expression to maximize ECs outputs for multi-pathways CLI therapy. Chem. Eng. J..

[232] Wang X., Gao B., Suleiman G.S.A., Ren X.-k., Guo J., Xia S., Zhang W., Feng Y. (2021). A “controlled CO release” and “pro-angiogenic gene” dually engineered stimulus-responsive nanoplatform for collaborative ischemia therapy. Chem. Eng. J..

[233] Wang X., Gao B., Ren X.-k., Guo J., Xia S., Zhang W., Yang C., Feng Y. (2021). A two-pronged approach to regulate the behaviors of ECs and SMCs by the dual targeting-nanoparticles. Colloids Surf. B-Biointerfaces.

[234] Wang X., Gao B., Wang M., Wang Q., Xia S., Zhang W., Meng X., Feng Y. (2023). CO delivery nanosystem based on regenerative bioactive zinc MOFs highlights intercellular crosstalk for enhanced vascular remodeling in CLI therapy. Chem. Eng. J..

[235] Gao B., Wang X., Wang M., You K., Suleiman G.S.A., Ren X.-K., Guo J., Xia S., Zhang W., Feng Y. (2022). Superlow Dosage of Intrinsically Bioactive Zinc Metal-Organic Frameworks to Modulate Endothelial Cell Morphogenesis and Significantly Rescue Ischemic Disease. ACS Nano.

[236] Hao X., Gai W., Ji F., Wang L., Zhao J., Yang F., Jiang H., Feng Y. (2022). Biomimetic and responsive nanoparticles loading JQ1 for dual-targeting treatment of vascular restenosis via multiple actions. Chem. Eng. J..

[237] Zhao J., Li Y., Wang M., Chen X., Kong D., Wang K., Feng Y. (2022). Oligoglycine and fluoropolymer functionalized enzyme-responsive gene delivery surface for rapid in situ endothelialization of vascular grafts. Appl. Mater. Today.

[238] Wang X., Gao B., Feng Y. (2022). Recent advances in inhibiting atherosclerosis and restenosis: From pathogenic factors, therapeutic molecules to nano-delivery strategies. J. Mater. Chem. B.

[239] Wang X., Gao B., Zhou J., Ren X.-k., Guo J., Xia S., Zhang W., Feng Y. (2020). Unexpected Amplification of Synergistic Gene Expression to Boom Vascular Flow in Advantageous Dual-Gene Co-expression Plasmid Delivery Systems over Physically Mixed Strategy. Acs Appl. Bio Mater..

[240] Gao B., Wang X., Wang M., Ren X.-k., Guo J., Xia S., Zhang W., Feng Y. (2020). From single to a dual-gene delivery nanosystem: Coordinated expression matters for boosting the neovascularization in vivo. Biomater. Sci..

[241] Li Q., Hao X., Wang H., Guo J., Ren X.-k., Xia S., Zhang W., Feng Y. (2019). Multifunctional REDV-G-TAT-G-NLS-Cys peptide sequence conjugated gene carriers to enhance gene transfection efficiency in endothelial cells. Colloids Surf. B-Biointerfaces.

[242] Gao B., Zhang Q., Wang X., Wang M., Ren X.-k., Guo J., Xia S., Zhang W., Feng Y. (2019). A “self-accelerating endosomal escape” siRNA delivery nanosystem for significantly suppressing hyperplasia via blocking the ERK2 pathway. Biomater. Sci..

[243] Hao X., Li Q., Wang H., Muhammad K., Guo J., Ren X., Shi C., Xia S., Zhang W., Feng Y. (2018). CAGW Modified Polymeric Micelles with Different Hydrophobic Cores for Efficient Gene Delivery and Capillary-like Tube Formation. Acs Biomater. Sci. Eng..

[244] Chen K., Tong C., Yang J., Cong P., Liu Y., Shi X., Liu X., Zhang J., Zou R., Xiao K. (2021). Injectable melatonin-loaded carboxymethyl chitosan (CMCS)-based hydrogel accelerates wound healing by reducing inflammation and promoting angiogenesis and collagen deposition. J. Mater. Sci. Technol..

[245] Sun X., Jia P., Zhang H., Dong M., Wang J., Li L., Bu T., Wang X., Wang L., Lu Q. (2022). Green Regenerative Hydrogel Wound Dressing Functionalized by Natural Drug-Food Homologous Small Molecule Self-Assembled Nanospheres. Adv. Funct. Mater..

[246] Shao Z., Yin T., Jiang J., He Y., Xiang T., Zhou S. (2023). Wound microenvironment self-adaptive hydrogel with efficient angiogenesis for promoting diabetic wound healing. Bioact. Mater..

[247] Zhang Q., Pei Q., Yang J., Guo S., Yang A., Qian Y., Li C., Feng Q., Lv H., Zhou X. (2022). Vascularized nanocomposite hydrogel mechanically reinforced by polyelectrolyte-modified nanoparticles. J. Mater. Chem. B.

[248] Xu Z., Liu G., Zheng L., Wu J. (2022). A polyphenol-modified chitosan hybrid hydrogel with enhanced antimicrobial and antioxidant activities for rapid healing of diabetic wounds. Nano Res..

[249] Zhang K., Chen X., Li H., Feng G., Nie Y., Wei Y., Li N., Han Z., Han Z.-c., Kong D. (2020). A nitric oxide-releasing hydrogel for enhancing the therapeutic effects of mesenchymal stem cell therapy for hindlimb ischemia. Acta Biomater..

[250] Cheng N.-C., Lin W.-J., Ling T.-Y., Young T.-H. (2017). Sustained release of adipose-derived stem cells by thermosensitive chitosan/gelatin hydrogel for therapeutic angiogenesis. Acta Biomater..

[251] Datta S., Rameshbabu A.P., Bankoti K., Roy M., Gupta C., Jana S., Das A.K., Sen R., Dhara S. (2021). Decellularized bone matrix/oleoyl chitosan derived supramolecular injectable hydrogel promotes efficient bone integration. Mater. Sci. Eng. C-Mater. Biol. Appl..

[252] Han H.-W., Hou Y.-T., Hsu S.-h. (2019). Angiogenic potential of co-spheroids of neural stem cells and endothelial cells in injectable gelatin-based hydrogel. Mater. Sci. Eng. C-Mater. Biol. Appl..

[253] Zhao N., Yue Z., Cui J., Yao Y., Song X., Cui B., Qi X., Han Z., Han Z.-C., Guo Z. (2019). IGF-1C domain-modified hydrogel enhances therapeutic potential of mesenchymal stem cells for hindlimb ischemia. Stem Cell Res. Ther..

[254] Qin D., Zhang A., Wang N., Yao Y., Chen X., Liu Y. (2022). Hydroxybutyl chitosan/oxidized glucomannan self-healing hydrogels as BMSCs-derived exosomes carriers for advanced stretchable wounds. Appl. Mater. Today.

[255] Zhang K., Zhao X., Chen X., Wei Y., Du W., Wang Y., Liu L., Zhao W., Han Z., Kong D. (2018). Enhanced Therapeutic Effects of Mesenchymal Stem Cell-Derived Exosomes with an Injectable Hydrogel for Hindlimb Ischemia Treatment. Acs Appl. Mater. Interfaces.

[256] Wang K., Dong R., Tang J., Li H., Dang J., Zhang Z., Yu Z., Guo B., Yi C. (2022). Exosomes laden self-healing injectable hydrogel enhances diabetic wound healing via regulating macrophage polarization to accelerate angiogenesis. Chem. Eng. J..

[257] Wu D., Qin H., Wang Z., Yu M., Liu Z., Peng H., Liang L., Zhang C., Wei X. (2022). Bone Mesenchymal Stem Cell-Derived sEV-Encapsulated Thermosensitive Hydrogels Accelerate Osteogenesis and Angiogenesis by Release of Exosomal miR-21. Front. Bioeng. Biotechnol..

[258] Jiang M., Pan Y., Liu Y., Dai K., Zhang Q., Wang J. (2022). Effect of sulfated chitosan hydrogel on vascularization and osteogenesis. Carbohydr. Polym..

[259] Yang X., Guo J.L., Han J., Si R.J., Liu P.P., Zhang Z.R., Wang A.M., Zhang J. (2020). Chitosan hydrogel encapsulated with LL-37 peptide promotes deep tissue injury healing in a mouse model. Mil. Med. Res..

[260] Li H., Li M., Liu P., Wang K., Fang H., Yin J., Zhu D., Yang Q., Gao J., Ke Q. (2021). A multifunctional substance P-conjugated chitosan hydrochloride hydrogel accelerates full-thickness wound healing by enhancing synchronized vascularization, extracellular matrix deposition, and nerve regeneration. Biomater. Sci..

[261] Aleem A.R., Shahzadi L., Tehseen S., Alvi F., Chaudhry A.A., Rehman I.U., Yar M. (2019). Amino acids loaded chitosan/collagen based new membranes stimulate angiogenesis in chorioallantoic membrane assay. Int. J. Biol. Macromol..

[262] Li Z., Fan X., Luo Z., Loh X.J., Ma Y., Ye E., Wu Y.-L., He C., Li Z. (2022). Nanoenzyme-chitosan hydrogel complex with cascade catalytic and self-reinforced antibacterial performance for accelerated healing of diabetic wounds. Nanoscale.

[263] Shen T., Dai K., Yu Y., Wang J., Liu C. (2020). Sulfated chitosan rescues dysfunctional macrophages and accelerates wound healing in diabetic mice. Acta Biomater..

[264] Haugen H.J., Lyngstadaas S.P., Rossi F., Perale G. (2019). Bone grafts: Which is the ideal biomaterial?. J. Clin. Periodontol..

[265] Zheng Z., Bian S., Li Z., Zhang Z., Liu Y., Zhai X., Pan H., Zhao X. (2020). Catechol modified quaternized chitosan enhanced wet adhesive and antibacterial properties of injectable thermo-sensitive hydrogel for wound healing. Carbohydr. Polym..

[266] Wu X., Li H. (2021). Incorporation of Bioglass Improved the Mechanical Stability and Bioactivity of Alginate/Carboxymethyl Chitosan Hydrogel Wound Dressing. ACS Appl. Bio Mater..

[267] Sheng L., Zhang Z., Zhang Y., Wang E., Ma B., Xu Q., Ma L., Zhang M., Pei G., Chang J. (2021). A novel “hot spring”-mimetic hydrogel with excellent angiogenic properties for chronic wound healing. Biomaterials.

[268] Zhang X., Huang P., Jiang G., Zhang M., Yu F., Dong X., Wang L., Chen Y., Zhang W., Qi Y. (2021). A novel magnesium ion-incorporating dual-crosslinked hydrogel to improve bone scaffold-mediated osteogenesis and angiogenesis. Mater. Sci. Eng. C-Mater. Biol. Appl..

[269] Chen Y., Sheng W., Lin J., Fang C., Deng J., Zhang P., Zhou M., Liu P., Weng J., Yu F. (2022). Magnesium Oxide Nanoparticle Coordinated Phosphate-Functionalized Chitosan Injectable Hydrogel for Osteogenesis and Angiogenesis in Bone Regeneration. Acs Appl. Mater. Interfaces.

[270] Qi X., Xiang Y., Cai E., You S., Gao T., Lan Y., Deng H., Li Z., Hu R., Shen J. (2022). All-in-one: Harnessing multifunctional injectable natural hydrogels for ordered therapy of bacteria-infected diabetic wounds. Chem. Eng. J..

[271] Tehrani F.D., Shabani I., Shabani A. (2022). A hybrid oxygen-generating wound dressing based on chitosan thermosensitive hydrogel and decellularized amniotic membrane. Carbohydr. Polym..

[272] Jiang L., Wang Y., Liu Z., Ma C., Yan H., Xu N., Gang F., Wang X., Zhao L., Sun X. (2019). Three-Dimensional Printing and Injectable Conductive Hydrogels for Tissue Engineering Application. Tissue Eng. Part B-Rev..

[273] Deng Z., Hu T., Lei Q., He J., Ma P.X., Guo B. (2019). Stimuli-Responsive Conductive Nanocomposite Hydrogels with High Stretchability, Self-Healing, Adhesiveness, and 3D Printability for Human Motion Sensing. Acs Appl. Mater. Interfaces.

[274] Li Y., Wang J., Wang Y., Cui W. (2021). Advanced electrospun hydrogel fibers for wound healing. Compos. Part B-Eng..

[275] Ferreira C.A.M., Januario A.P., Felix R., Alves N., Lemos M.F.L., Dias J.R. (2021). Multifunctional Gelatin/Chitosan Electrospun Wound Dressing Dopped with Undaria pinnatifida Phlorotannin-Enriched Extract for Skin Regeneration. Pharmaceutics.

[276] Russell G.M., Inamori D., Masai H., Tamaki T., Terao J. (2019). Luminescent and mechanical enhancement of phosphorescent hydrogel through cyclic insulation of platinum-acetylide crosslinker. Polym. Chem..

[277] Chang A., Babhadiashar N., Barrett-Catton E., Asuri P. (2020). Role of Nanoparticle-Polymer Interactions on the Development of Double-Network Hydrogel Nanocomposites with High Mechanical Strength. Polymers.

[278] Cai S., Lei T., Bi W., Sun S., Deng S., Zhang X., Yang Y., Xiao Z., Du H. (2022). Chitosan Hydrogel Supplemented with Metformin Promotes Neuron-like Cell Differentiation of Gingival Mesenchymal Stem Cells. Int. J. Mol. Sci..

[279] Re F., Sartore L., Moulisova V., Cantini M., Almici C., Bianchetti A., Chinello C., Dey K., Agnelli S., Manferdini C. (2019). 3D gelatin-chitosan hybrid hydrogels combined with human platelet lysate highly support human mesenchymal stem cell proliferation and osteogenic differentiation. J. Tissue Eng..

[280] Zhang Y., Li Y., Liu C., Shang X. (2022). Decellularized Nucleus Pulposus Matrix/Chitosan Hybrid Hydrogels for Nucleus Pulposus Tissue Engineering. Glob. Spine J..

[281] Ahtzaz S., Waris T.S., Shahzadi L., Chaudhry A.A., Rehman I.U., Yar M. (2020). Boron for tissue regeneration-it’s loading into chitosan/collagen hydrogels and testing on chorioallantoic membrane to study the effect on angiogenesis. Int. J. Polym. Mater. Polym. Biomater..

[282] Dieu Linh T., Phuong Le T., Thai Thanh Hoang T., Park K.D. (2020). Novel enzymatically crosslinked chitosan hydrogels with free-radical-scavenging property and promoted cellular behaviors under hyperglycemia. Prog. Nat. Sci.-Mater. Int..

[283] Liu Y., Wong C.-W., Chang S.-W., Hsu S.-h. (2021). An injectable, self-healing phenol-functionalized chitosan hydrogel with fast gelling property and visible light-crosslinking capability for 3D printing. Acta Biomater..

[284] Yu H., Liu J., Zhao Y.-Y., Jin F., Dong X.-Z., Zhao Z.-S., Duan X.-M., Zheng M.-L. (2019). Biocompatible Three-Dimensional Hydrogel Cell Scaffold Fabricated by Sodium Hyaluronate and Chitosan Assisted Two-Photon Polymerization. ACS Appl. Bio Mater..

